# Targeting autophagy and plasminogen activator inhibitor-1 increases survival and remodels the tumor microenvironment in glioblastoma

**DOI:** 10.1186/s13046-025-03473-w

**Published:** 2025-07-19

**Authors:** Sophie G. Shifman, Jennifer L. O’Connor, Daniel P. Radin, Aryan Sharma, Laura Infante, Francesca Ferraresso, Christian J. Kastrup, Daniel A. Lawrence, Stella E. Tsirka

**Affiliations:** 1 Molecular and Cellular Pharmacology Graduate Program, Department of Pharmacological Sciences, Renaissance School of Medicine at Stony Brook University, Stony Brook, NY USA; 2 Medical Scientist Training Program, Renaissance School of Medicine at Stony Brook University, Stony Brook, NY USA; 3 Program in Neuroscience, Stony Brook University, Stony Brook, NY USA; 4 Program in Physiology and Biophysics, Renaissance School of Medicine at Stony Brook University, Stony Brook, NY USA; 5 Versiti Blood Research Institute, Milwaukee, WI USA; 6 Department of Biochemistry and Molecular Biology, University of British Columbia, Vancouver, Canada; 7 Departments of Surgery, Biomedical Engineering, and Pharmacology and Toxicology, Medical College of Wisconsin, Milwaukee, WI USA; 8 Division of Cardiovascular Medicine, Department of Internal Medicine, University of Michigan Medical School, Ann Arbor, MI USA

## Abstract

**Background:**

Glioblastoma (GBM), the most common and aggressive type of primary brain tumor, engages multiple survival mechanisms, including autophagy. GBM exploits both degradative and secretory autophagy pathways to support tumor growth and limit the efficacy of standard-of-care treatments. We have previously shown that lucanthone, a blood-brain barrier permeable autophagy inhibitor, reduces tumor burden. However, although lucanthone-treated tumors are significantly smaller in size, they are not completely obliterated, suggesting compensatory survival mechanisms. A critical factor for GBM survival is communication with the tumor microenvironment (TME), which can be programmed by glioma cells to support growth and immunosuppression. Plasminogen activator inhibitor-1 (PAI-1), a secreted serine protease inhibitor, has been implicated in the progression of several cancers, including GBM, and has been shown to be modulated by autophagy in other cancers. The role of PAI-1 in GBM, namely its relationship with intracellular autophagy dysregulation and extracellular TME as a mechanism of tumor survival, remains incompletely understood.

**Methods:**

Murine glioma models were established using intracranial injection of GL261 cells in C57BL/6 mice, followed by autophagy inhibition with intraperitoneal lucanthone and/or PAI-1 inhibition with MDI-2268 chow, and tumors were assessed by immunohistochemistry. In culture, glioma cell lines were challenged with MDI-2268, lucanthone, mitoxantrone, or siRNA-LNPs targeting PAI-1, and assessed by MTT assay, q-RT-PCR, ELISA, invasion assay, immunoblot, and immunocytochemistry. Lysosomal markers and transient transfection with fluorescent vesicular proteins were utilized to evaluate PAI-1 intracellular localization via confocal microscopy. Synergy was analyzed using the HSA model in Combenefit, and statistical analyses included t-tests, ANOVA, and log-rank tests for survival.

**Results:**

Lucanthone treatment increased intracellular PAI-1 and autophagy markers while reducing active extracellular PAI-1. PAI-1 colocalized with lysosomal markers, suggesting impaired secretory autophagy. PAI-1 inhibition reduced glioma cell viability and invasion. Combination therapy with lucanthone and MDI-2268 drastically decreased tumor volume, prolonged survival, and promoted a pro-inflammatory state in the tumor microenvironment.

**Conclusions:**

Our findings suggest that PAI-1 may be a compensatory survival mechanism in GBM after autophagy inhibition, and that dual targeting of autophagy and PAI-1 disrupts tumor progression and enhances anti-tumor immunity, providing promising evidence for targeting this axis.

**Supplementary Information:**

The online version contains supplementary material available at 10.1186/s13046-025-03473-w.

## Introduction

Glioblastoma (GBM) is a primary adult brain tumor characterized by heterogeneity, aggressiveness, and recurrence. It is the most frequent and malignant form of primary brain tumor and comprises up to 50% of all gliomas [1]. Despite advancements in treatment modalities, median survival for patients with GBM is 15 months and less than 5% of patients survive 5 years post-diagnosis [1]. Prognosis remains poor due to failure of standard of care, which is maximal tumor resection followed by radiation and temozolomide treatment, and which has remained virtually unchanged for the past twenty years [2, 3]. Presently, recurrence of GBM is inevitable, and usually arises immediately next to the resected area [1–3]supporting the notion that existing therapies are not addressing key components of this unrelenting, heterogenous, and resourceful tumor. Thus, there continues to be a desperate need for improvement of treatments for this dismal disease.

GBM employs a battery of survival mechanisms that help cells grow uncontrollably and resist treatment. One of the many avenues that GBM takes to accomplish this goal is cytoprotective autophagy. This is a vital intracellular process that both recycles cellular components through lysosomal degradation, known as degradative autophagy, and participates in secretion of factors to the extracellular space, known as secretory autophagy [4]. Autophagy is implicated in promoting tumor cell survival, especially as the tumor is subjected to stress conditions prompted by nutrient deprivation and hypoxia, which frequently occurs in GBM [5–7]. GBM takes advantage of autophagy to persist and this limits the efficacy of standard-of-care treatments. Our laboratory previously described the effects of lucanthone, a brain-penetrable inhibitor of autophagy, on GBM survival and growth [8, 9]. Through autophagy blockade, lucanthone administration slows GBM growth and normalizes tumor vasculature [8, 9]. As our studies show, however, lucanthone-treated tumors are substantially decreased in volume, but are not entirely eradicated. Moreover, the landscape that emerges as a response to lucanthone treatment has not been characterized.

An important aspect in GBM survival is communication with and manipulation of the brain tumor microenvironment (TME), which is made up of a host of different cell types that can be hijacked by cancer cells to promote an immunosuppressive niche that supports tumor progression [10]. In a concerted effort to overcome GBM, one of the strategies is to repolarize the TME towards a more pro-inflammatory, or immune-stimulatory, state that will result in decreased tumor growth [10–13].

Plasminogen activator inhibitor-1 (PAI-1, or SERPINE1) is a member of the serine protease inhibitor (SERPIN) superfamily. It is a secreted factor that is expressed by nearly all cell types. In addition to its role in inhibition of fibrinolysis, PAI-1 exhibits multiple other functions, including tumor progression [14, 15]. PAI-1 plays an important role in several cancer contexts, including melanoma, breast, lung, fibrosarcoma, prostate, and glioma [16–27]. The function of PAI-1 in GBM, and specifically its role intracellularly in autophagy and extracellularly in modulating the TME, remains incompletely understood. We set out to investigate this role of PAI-1, and how it may be exploited to improve therapy for GBM. In this study, we identify PAI-1 as a critical factor mediating GBM response to autophagy inhibition by lucanthone. We demonstrate that blocking PAI-1, using the newly described specific inhibitor MDI-2268 [36], slowed glioma cell growth. Additionally, we observed that PAI-1 participated in the autophagy pathway in GBM and that PAI-1 secretion was partially autophagy dependent. Furthermore, dual PAI-1-and autophagy- inhibited tumors were significantly smaller, their microenvironment comprised activated immune cells, and their animal hosts lived longer. These findings highlight a potential mechanism through which GBM can exploit PAI-1 to resist treatment and support further investigation into therapeutic strategies targeting this pathway.

## Materials and methods

### Cell culture

Glioma cell lines were described previously [9]. Briefly GL261-FmC (Firefly luciferase + mCherry+) cells were a kind gift from Dr. Khalid Shah’s lab. GL261 cell line is derived from a chemically induced astrocytoma in C57BL/6 mice [28]. KR158 cells were obtained from the labs of Drs. Tyler Jacks and Behnam Badie and are derived from genetically engineered Nf1/Tp53 mutants [29]. Two primary patient-derived glioma cell lines were also utilized in some of these studies: GBM43 and GBM9, which carry Nf1 and Tp53 mutations and Kras and Tp53 mutations, respectively. The lines were obtained from the Mayo Clinic Brain Tumor Patient-Derived Xenograft National Resource. Cells were maintained in DMEM, 10% serum, 1% antibiotic, 1% sodium pyruvate and incubated at 37 °C with 5% CO_2_. To enrich for glioma stem-like cells (GSC) in GL261 adherent cells, serum was reduced stepwise over a week as described previously [9]. GSC were cultured in serum-free DMEM medium containing F12 supplement along with pyruvate, antibiotics, N2 supplement, epidermal growth factor, fibroblast growth factor, and heparin [9]. BV2 and N9 mouse microglia cell lines, described previously [30] were utilized in conditioned media experiments.

### RNA extraction and quantitative RT-PCR (qPCR)

RNA was isolated from cells using the Qiagen RNeasy Mini Kit according to manufacturer’s protocol. To obtain cDNA, one microgram of RNA was reverse transcribed on a Veriti thermocycler using the High Capacity cDNA Reverse Transcription Kit. Amplification was performed on a StepOnePlus real-time PCR machine using a SYBR green kit (Applied Biosystems). Primer sequences are as follows: GAPDH forward, 5′-GCACAGTCAAGGCCGAGAAT-3′; GAPDH reverse, 5′-GCCTTCTCCATGGTGGTGGA-3′; PAI-1 forward, 5′-CAATGGAAGGGCAACATGACC-3′; PAI-1 reverse, 5′-AGCTGCTCTTGGTCGGAAA-3′. GAPDH was used as an internal control.

### MTT assay

Cells were plated in a 96-well plate at 3 or 5 × 10^3^ cells per well and incubated overnight, then treated with DMSO control and/or MDI-2268 and/or lucanthone solubilized in DMSO, or siRNA-LNPs, at the indicated concentrations and time points, and then subjected to the MTT protocol, per manufacturer’s instructions (Sigma-Aldrich 475989). Briefly, MTT powder was solubilized at 5 mg/ml in 1X HBSS, 10 µL was added to each well, and incubated for 4 h at 37 °C. 100 µL of stop solution (1 N HCL, 10% SDS, in diH_2_O) was added to each well and incubated overnight. Plates were subsequently read at 570 and 690 nm, and 690 absorbance values (background) were subtracted from 570 absorbance values. Control values were averaged and all values converted to percentage of the average of control (% relative live cell count).

### Immunocytochemistry

For immunocytochemical analysis, cells were plated on glass coverslips overnight, and then treated with the indicated drugs and time points. Cells were fixed with 4% PFA for 10 min, then washed 3x with 0.3% TX-100 in PBS and wells were blocked with 10% normal goat or donkey serum diluted in 0.3% TX-100 in PBS for 1 h. Cells were stained with primary antibodies overnight at 4 °C (see Suppl. Table 1). The primary antibody was removed, and cells were again washed 3x with 0.3% TX-100 in PBS, after which cells were incubated with appropriate fluorescent secondary antibodies (Alexa Fluor) for 1 h at room temperature. Cells were then washed 3x with 0.3% TX-100 in PBS, mounted onto glass slides using DAPI Fluoromount counterstain (Southern Biotech 0100 − 20), and imaged under the Leica SP8-x confocal microscopy system, with white light and argon lasers. Quantifications were performed using FIJI (ImageJ), as the measured integrated density of each channel divided by the number of DAPI + nuclei in each field of view, i.e. per cell. Colocalization was determined as the overlapping integrated density of the colocalizing channels.

### ELISA

To investigate active and total PAI-1 extracellular secretion, ELISA assays were performed on cell culture supernatants, per manufacturer’s instructions (Innov Res IMSPAI1KTA, IMSPAI1KTT). Briefly, media were taken from treated cells, spun down to pellet any dead cells or debris, supernatant was transferred to new tubes, and either placed on ice or frozen at -80 °C until ELISA could be performed, at which point samples were thawed on ice and either diluted further with diH2O or not. 100 µL of each sample was used in the assay.

### Overexpression of Rab GTPases

For transient overexpression of target Rab GTPases conjugated to a fluorescent reporter, cells were plated onto coverslips, then transfected with Rab7a-GFP, Rab5-GFP, Rab27a-GFP, or Rab27b-GFP plasmid DNA, in triplicate, at 500 ng per well each for 48 h, using Lipofectamine 3000 transfection reagent (Invitrogen L3000001) according to manufacturer’s protocol. Cells were subsequently treated with 10 µM lucanthone for an additional 24 h to induce PAI-1, fixed and stained with PAI-1 and LAMP1 primary antibodies, appropriate secondary antibodies, counterstained with DAPI and mounted onto slides, and imaged by confocal microscopy.

### siRNA-LNP PAI-1 formulation and knockdown

siRNA targeting PAI-1 lipid nanoparticles (LNPs) were prepared as described previously [31, 32]. Briefly, siRNA was dissolved in sodium acetate (pH 4) and rapidly mixed with a lipid solution consisting of ALC-0315, DSPC, cholesterol, and PEG-DMG (Avanti Polar Lipids, Birmingham, AL) at a 50:10:38.5:1.5% molar ratio. The LNPs were allowed to dialyze overnight in phosphate-buffered saline (PBS) at pH 7.4 and diluted to a final concentration of 0.1 mg/mL. Cells were incubated with either control or anti-PAI-1 LNPs diluted in media for 72 h and either evaluated by western blot to confirm PAI-1 knockdown or by MTT assay for cell viability.

### Invasion assay

Invasion assay was done as described previously [21]. Briefly, 8 μm transwell chambers in a 24-well plate were pre-coated with Matrigel, and 1 × 10^5^ GL261 cells pre-mixed in serum-free medium with either DMSO control, 2.5 µM MDI-2268, 10 µM lucanthone, or the combination were added to each transwell. The lower chamber of each well was filled with medium containing 10% serum. After 24 h of incubation, non-migrated cells on the upper side of the transwell membranes were removed using a cotton swab. The bottom side of the membrane was washed with PBS, incubated with cold methanol for 20 min to fix the cells that crossed to the bottom side of the membrane, stained with 0.1% Crystal Violet, and imaged using a light microscope under 10x magnification. To quantify the Crystal Violet staining, the wells were incubated with 10% acetic acid to dissolve the staining, the transwell inserts were removed, and the plate was read at 595 nm using a microplate reader.

### Animals

C57BL/6 (WT) mice were housed on a 12:12 h light: dark cycle with regular mouse chow ad libitum.

### Murine glioma model

Gliomas were established in immunocompetent 6-month-old male and female mice as described previously [33–35]. Mice were anesthetized using inhaled isoflurane and kept on a steady flow throughout the procedure, a midline incision was made in the scalp, the skin retracted and a small burr hole was drilled in the skull. 1 × 10^5^ GL261-FmC resuspended in PBS were injected over a period of 2 min at a depth of 3 mm. At the end of the injection, the needle was kept in the injection site for a further 4 min. After needle removal, the incision was sutured and mice were placed on a heating pad until they fully recovered from anesthesia. During the disease course, if mice were found to have lost more than 15% of their initial body weight, or if, due to the size or location of the tumor, they could not move normally or easily reach food and water, they were euthanized. All animal procedures were carried out in strict accordance with and approved by the Stony Brook University Institutional Animal Care and Use Committee.

### MDI-2268 and lucanthone treatment in vivo

Upon establishment of detectable glioma growth, mice were either placed on control chow or chow containing 500 mg of MDI-2268 per kg of chow [36–38] which was obtained from Dr. Daniel Lawrence’s lab (University of Michigan Medical School). Lucanthone was supplied by Dr. Robert Bases (Albert Einstein College of Medicine). Lucanthone was solubilized in 10% DMSO, 40% 2-Hydroxypropyl-β-cyclodextrin in PBS. Mice were randomly divided into control and experimental groups, and treated with either saline or 50 mg/kg lucanthone i.p. daily throughout the remainder of the study.

### Immunohistochemistry

Mice were anesthetized with 20 mg/kg avertin and transcardially perfused with 30 mL of cold PBS followed by 30 mL of cold 4% PFA in PBS. Brains were removed and post-fixed in 4% PFA in PBS overnight. After 24 h, they were embedded in 30% w/v sucrose in PBS to dehydrate and cryo-preserve them. Brains were then embedded in optimal cutting temperature compound (OCT, Tissue-Tek) and 20 μm coronal sections throughout the tumor were taken on a Leica cryostat (Nusslock, Germany) and collected on Superfrost plus microscope slides. To determine tumor volume, serial sections were taken from each animal and subjected to hematoxylin and eosin (H&E) stain. Tumor volume was calculated as tumor area x 20 μm thickness, x number of slides [39]. For immunohistochemical analysis, slides were washed 3x with 0.3% TX-100 in PBS and then blocked with 10% normal goat or donkey serum diluted in 0.3% TX-100 in PBS for 1 h. Slides were incubated overnight at 4 °C with appropriate primary antibodies (see Suppl. Table 1). The primary antibody was removed and slides were washed 3x with 0.3% TX-100 in PBS and incubated with appropriate fluorescent secondary antibodies (Alexa Fluor) for 1 h. Slides were washed 3x with 0.3% TX-100 in PBS, counterstained with DAPI, and visualized by confocal imaging using the Leica SP8-x system. Quantifications were performed using FIJI (ImageJ), as the measured integrated density of each channel either per field of view or divided by the number of DAPI + nuclei in each field of view, i.e. per cell. Colocalization was determined as the overlapping integrated density of the colocalizing channels.

### Western blot

Immunoblotting was done as described previously [9]. Briefly, cells were lysed in 50 mM Tris-HCl (pH 7.4) with 1% Nonidet P- 40, 0.25% sodium deoxycholate, 150 mM NaCl, 1% SDS and 1 mM sodium orthovanadate. Proteins were denatured by boiling and treatment with β-mercaptoethanol. Proteins were run on SDS-page gels and transferred onto PVDF membranes (Immobilon; Millipore). Membranes were washed with Tris-buffered saline with 0.1% Tween 20 (TBS-T) and blocked in a 5% BSA in TBS-T solution for 1 h. Membranes were then incubated with appropriate primary antibodies overnight at 4 °C (see Suppl. Table 1). Membranes were rinsed in TBS-T, probed with associated HRP-conjugated secondary antibodies and exposed to SuperSignal West Pico PLUS Chemiluminescent Substrate for 1 min (Thermo Fisher Scientific), and subsequently developed using the iBright Imaging System. Quantifications were performed using FIJI (ImageJ).

### Synergy analysis

Synergy analysis of the combined effect of MDI-2268 and lucanthone on cell viability was performed using the Combenefit software [40] wherein the HSA model was used.

### Patient data analysis

All GBM patient survival and gene correlation data was plotted and analyzed using the Gliovis online database and the TCGA_GBM dataset.

### Statistical analysis

See Supplementary Table 2 for all statistical analyses performed in this study. Briefly, data comparing two population means with a normal distribution were analyzed using unpaired t-test. One- and two-way ANOVA and Tukey’s test were used to compare more than two populations. Log-rank (Mantel-Cox) tests were used in survival analysis to compare individual populations. In all plots, error bars represent mean +/- standard deviation (SD). Alpha value was set at 0.05 prior to starting experiments. In all significance analyses, * signifies a p-value < 0.05, ** = *p* < 0.01, *** = *p* < 0.001, and **** = *p* < 0.0001. Power analysis was used to determine the appropriate number of animals used in each experiment. Statistical analysis was performed using Graphpad Prism (Graphpad Software Inc, La Jolla, CA).

## Results

### PAI-1 expression correlates with poor GBM prognosis and is increased in tumors treated with lucanthone

**Fig. 1:**
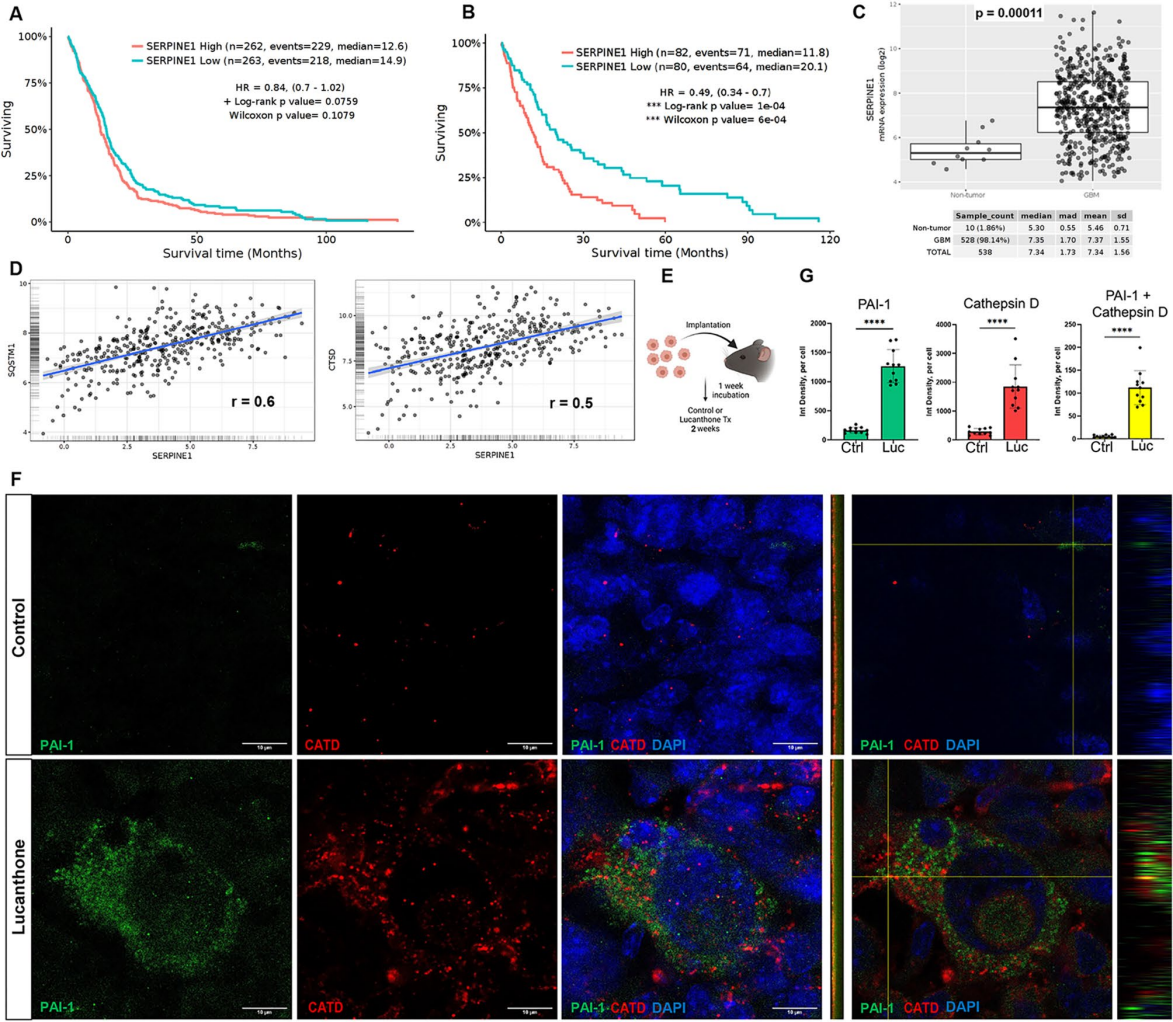
PAI-1 expression correlates with poor GBM prognosis and is increased in tumors treated with lucanthone. (**A**) Kaplan-Meier survival curve of GBM patients with tumors of all subtypes that express low or high levels of PAI-1 (SERPINE1) (Gliovis/TCGA_GBM dataset). (**B**) Kaplan-Meier survival curve of GBM patients with tumors of the proneural subtype that express low or high levels of PAI-1 (SERPINE1) (Gliovis/TCGA_GBM dataset). (**C**) mRNA expression of PAI-1 (SERPINE1) in GBM compared to non-tumor tissue, unpaired t-test, p-value = 0.00011 (Gliovis/TCGA_GBM dataset using the HG-U133A platform). (**D**) Correlation plots of PAI-1 expression vs. expression of autophagy markers p62 (SQSTM1) and Cathepsin D (CTSD) in GBM tumors, labeled with respective Pearson correlation coefficients (Gliovis). (**E**) Cartoon of in vivo mouse GBM model detailing implantation of GL261 cells and treatment course. (**F**) Representative confocal images of tumors treated with saline control or lucanthone for two weeks and stained with PAI-1, Cathepsin D (CATD), and DAPI nuclear stain. Colocalization of PAI-1 and CATD are shown in the reslice and orthogonal views. Scale bars measure 10 μm. (**G**) Quantification of the signal integrated density divided by the number of DAPI + nuclei, i.e. per cell, shown in F. Each dot represents one animal, average of 3–5 images per animal, *n* = 11 animals per group, unpaired t-tests

Upon examination of publicly available GBM datasets (Gliovis), a correlation was evident between elevated expression of PAI-1 (SERPINE1) and reduced survival times across all GBM patients. Specifically, patients with low PAI-1-expressing tumors demonstrated slightly longer median survival compared to those with high PAI-1 expression (Fig. 1A). This correlation was more pronounced within the proneural GBM subtype (Fig. 1B). Additionally, GBM tissue exhibited significantly higher PAI-1 mRNA expression compared to non-tumor brain tissue (Fig. 1C). Further analysis revealed a positive correlation between PAI-1 expression and autophagy markers p62 (SQSTM1) and Cathepsin D (CTSD) in GBM tissues (Fig. 1D), suggesting that PAI-1 may be modulating GBM tumor biology through the process of autophagy.

To further investigate this possibility, an in vivo murine GBM model of GL261 mouse glioma (Fig. 1E) was employed. GL261 cells were injected intracerebrally and allowed to grow for 7 days. Once a visible tumor was detected using bioluminescence imaging (BLI/IVIS), the animals were treated with the autophagy inhibitor lucanthone. Lucanthone administration resulted in significant increases in Cathepsin D, as expected, and in PAI-1 (Fig. 1F, G; Suppl. Figure 1). Immunofluorescence revealed colocalization of PAI-1 and Cathepsin D, but notable accumulation of PAI-1 was also observed outside these autophagic vesicles (Fig. 1F, G). Taken together, these findings suggest that PAI-1 may serve as a marker of GBM tumor progression and specifically, PAI-1 could be a cellular response to lucanthone treatment as a mechanism of survival against autophagy inhibition.

### Lucanthone increases autophagy markers and intracellular PAI-1 both within and outside lysosomal machinery

**Fig. 2:**
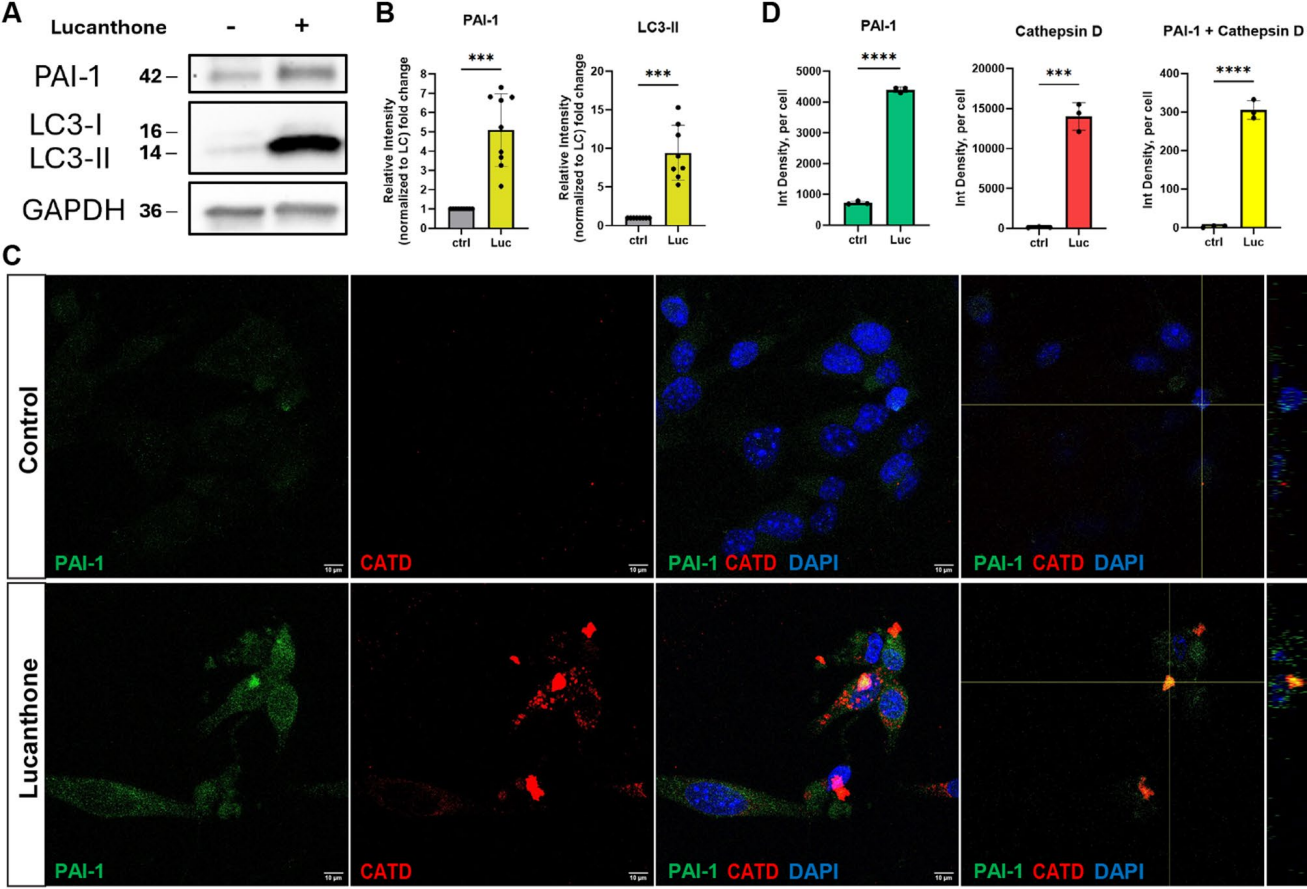
Lucanthone increases autophagy markers and intracellular PAI-1 both within and outside lysosomal machinery. (**A**) Representative western blot of PAI-1 and LC3 expression in GL261 cells treated with DMSO control or 10 µM lucanthone for 24 h. GAPDH was used as loading control. Numbers next to blots are kDa based on protein ladder. (**B**) Quantifications of PAI-1 and LC3-II signal intensity, normalized to loading control (LC). Data presented as fold change of ctrl (DMSO). Each dot represents one independent experiment, unpaired t-tests. (**C**) Representative images of GL261 cells treated with DMSO control or 10 µM lucanthone for 24 h in regular media, stained with PAI-1, Cathepsin D (CATD), and DAPI. Colocalization of PAI-1 and CATD are shown in orthogonal views. Scale bars measure 10 μm. (**D**) Quantification of C. Each dot represents one independent experiment, average of 3–5 images per experiment, unpaired t-tests

In culture experiments, immunoblot analysis of glioma cell extracts revealed that addition of 10µM lucanthone for 24 h resulted in increased levels of PAI-1 and the autophagy marker LC3-II (Fig. 2A, B). Moreover, lucanthone treatment also increased PAI-1 mRNA expression in these cells (Suppl. Figure 2A) and PAI-1 protein level in two patient-derived glioma cell lines [9] (Suppl. Figure 2B). These data suggest that PAI-1 and its response to lucanthone treatment may also be important in human GBM disease. Immunofluorescence confirmed elevated intracellular PAI-1 and Cathepsin D levels, showing widespread PAI-1 cellular distribution, including within autophagic vesicles (Fig. 2C, D).

To investigate where, beyond autophagic vesicles, PAI-1 localizes within the cell when autophagy is inhibited by lucanthone, other markers of endosomal and exosomal vesicles were utilized. Rab GTPases are highly conserved regulators of membrane and vesicular shuttling within cells [41]. Rab7 participates in late endosomal to lysosomal trafficking and regulates the maturation of the autophagosome, Rab5 is found in early endosomes, while Rab27 regulates exocytosis [41].

To determine the intracellular localization of PAI-1 following autophagy inhibition, we examined its colocalization with various Rab GTPases that mark distinct vesicular compartments. Rab7a, Rab5, Rab27a, or Rab27b conjugated to GFP, a fluorescent reporter, were transiently transfected (Suppl. Figure 3A-E). Cells were then treated with lucanthone and subsequently stained. Confocal imaging revealed that PAI-1 colocalized most strongly with Rab7a and LAMP1, markers of late endosomes and lysosomes, respectively, whereas colocalization with Rab5 (early endosomes) and Rab27a/b (exosomal pathways) was minimal (Suppl. Figure 3F). These findings suggest that PAI-1 is trafficked predominantly through the autophagy-lysosome pathway and accumulates in late endosomal and lysosomal compartments upon lucanthone treatment. This supports a model in which autophagy inhibition disrupts normal PAI-1 processing and secretion, leading to its intracellular retention.

### PAI-1 and autophagy inhibition decrease glioma cell proliferation, stemness, and invasion

PAI-1 knockdown by siRNA and shRNA has been shown to lead to defects in cancer cell migration, invasion, tube formation, and dispersal [21, 26]. To investigate the effect of knocking down PAI-1 on cell viability in our glioma model, an innovative siRNA approach, described previously [31, 32] was utilized. In this method, siRNA is encapsulated into lipid nanoparticles (LNPs) and delivered directly to a system, making the siRNA easily accessible in vitro and in vivo. Two mouse glioma cell lines, GL261 and KR158, were incubated with control siRNA LNPs or siRNA targeting mouse PAI-1 LNPs; PAI-1 knockdown was confirmed, and cell viability was measured. Knockdown of PAI-1 resulted in a significant decrease in the percentage of live cells relative to control in both cell lines (Suppl. Figure 4A, B).

**Fig. 3:**
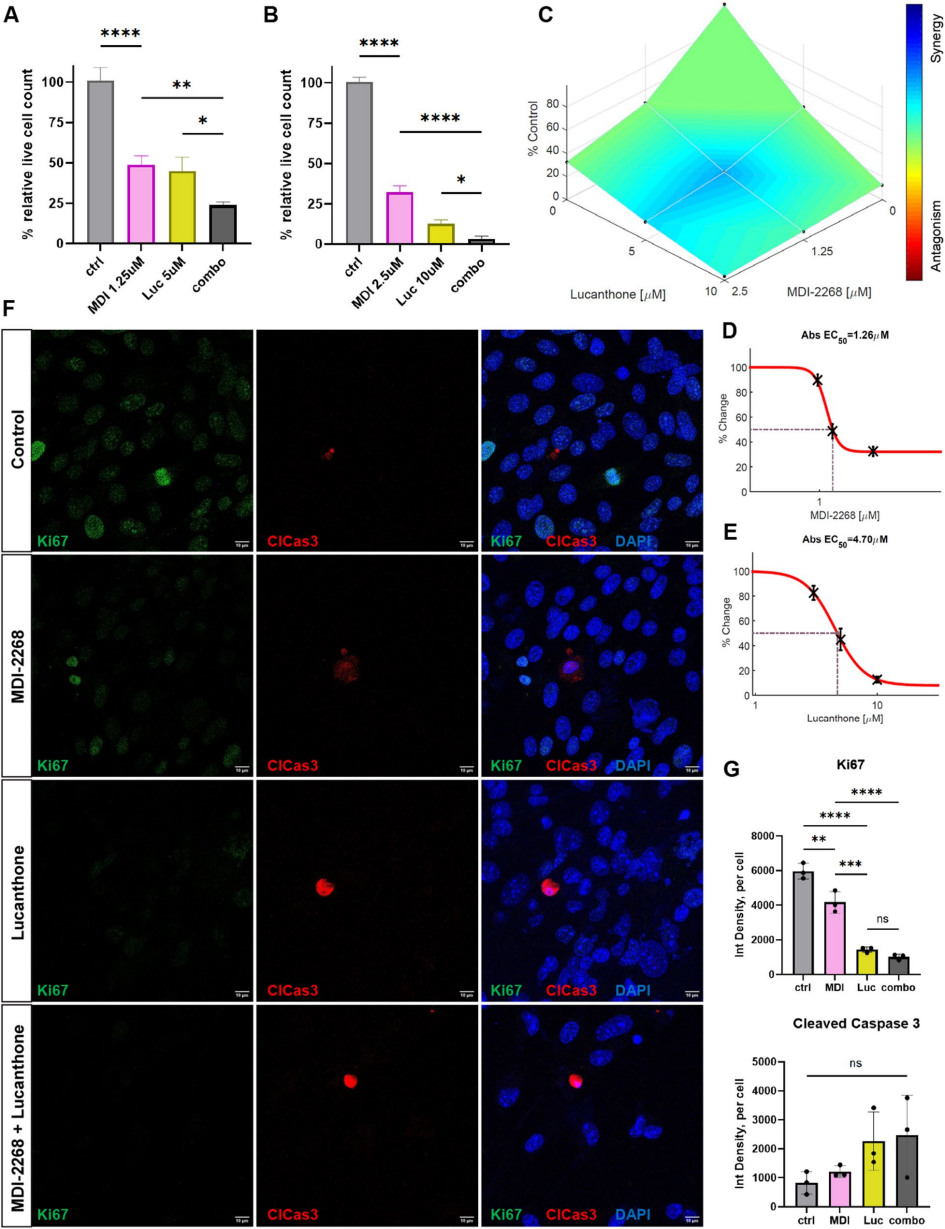
PAI-1 and autophagy inhibition decrease glioma cell proliferation. (**A, B**). MTT assay measuring percentage of live GL261 cells treated with DMSO control, 1.25 and 2.5 µM MDI-2268, 5 and 10 µM lucanthone, or the combination, respectively, for 72 h in regular media (10% serum) relative to the average of control. *n* = 3 independent experiments, one-way ANOVA. (**C**) HSA synergy analysis of the two agents using Combenefit software. (**D, E**) Cell viability dose-response curves for MDI-2268 and lucanthone, respectively, in GL261 cells, and their EC50 values (% change = % change in relative live cells) plotted using Combenefit. Each data point is the mean (marked with an X) and error bar for 3 independent experiment values. (**F**) Representative images of GL261 cells treated with DMSO control, 2.5 µM MDI-2268, 10 µM lucanthone, or the combination for 48 h in regular media (10% serum), and stained with Ki67, cleaved Caspase 3, and DAPI. Scale bars measure 10 μm. (**G**) Quantification of F, each dot represents one independent experiment, average of 3–5 images per experiment, one-way ANOVA

To investigate the effect of inhibiting PAI-1 on cell viability, MDI-2268, a selective inhibitor of PAI-1 that is orally bioavailable and brain-penetrable [36–38] was used. MDI-2268 inhibits PAI-1’s anti-protease activity when it is bound to vitronectin [36–38] a multifunctional protein that is found in blood and as part of the extracellular matrix [42]. To reveal the cell viability response to MDI-2268 compared with lucanthone in glioma, we incubated GL261 cells with increasing concentrations of either drug separately (Suppl. Figure 4C). Treatment with MDI-2268 significantly reduced glioma cell viability, which was further decreased by combining MDI-2268 with lucanthone (Fig. 3A, B). Synergy analysis confirmed that these agents act synergistically at lower concentrations (namely at 1.25 µM MDI-2268 and 5 µM lucanthone, Fig. 3C), and the observed EC50 values were 1.26 µM for MDI-2268 and 4.70 µM for lucanthone (Fig. 3D, E). The drug treatments significantly reduced cell proliferation (Ki67 expression), but apoptosis (cleaved Caspase 3) remained unaffected (Fig. 3F, G).

GBM consists of multiple tumor cell subpopulations in different stages of differentiation, including a stem-like subset known as glioma stem cells (GSC) characterized by self-renewal and tumor-initiating capabilities [43]. To investigate whether PAI-1 and autophagy inhibition influences stem-like properties of distinct populations of glioma cells, GSC, which grow in 3D spheroids, were treated and assessed in comparison with their adherent, or 2D monolayer, counterparts for changes in markers of stemness, such as Nestin, Olig2, and S100B [43, 44]. After 24 h, MDI-2268-treated GSC had substantially lower Olig2 and S100B expression, whereas combination-treated adherent glioma cells showed reduction in expression of all three proteins (Suppl. Figure 4D-G). Finally, an invasion assay at this timepoint demonstrated that MDI-2268, lucanthone, and combination treated glioma cells were all less invasive than control treated cells, with the combination showing the greatest reduction in invaded cells (Suppl. Figure 4H). Altogether, these experiments reveal the breadth of the impact of combining PAI-1 and autophagy inhibition in glioma with these two agents.

### Lucanthone and combination treatments increase intracellular PAI-1 but abolish active extracellular PAI-1

**Fig. 4:**
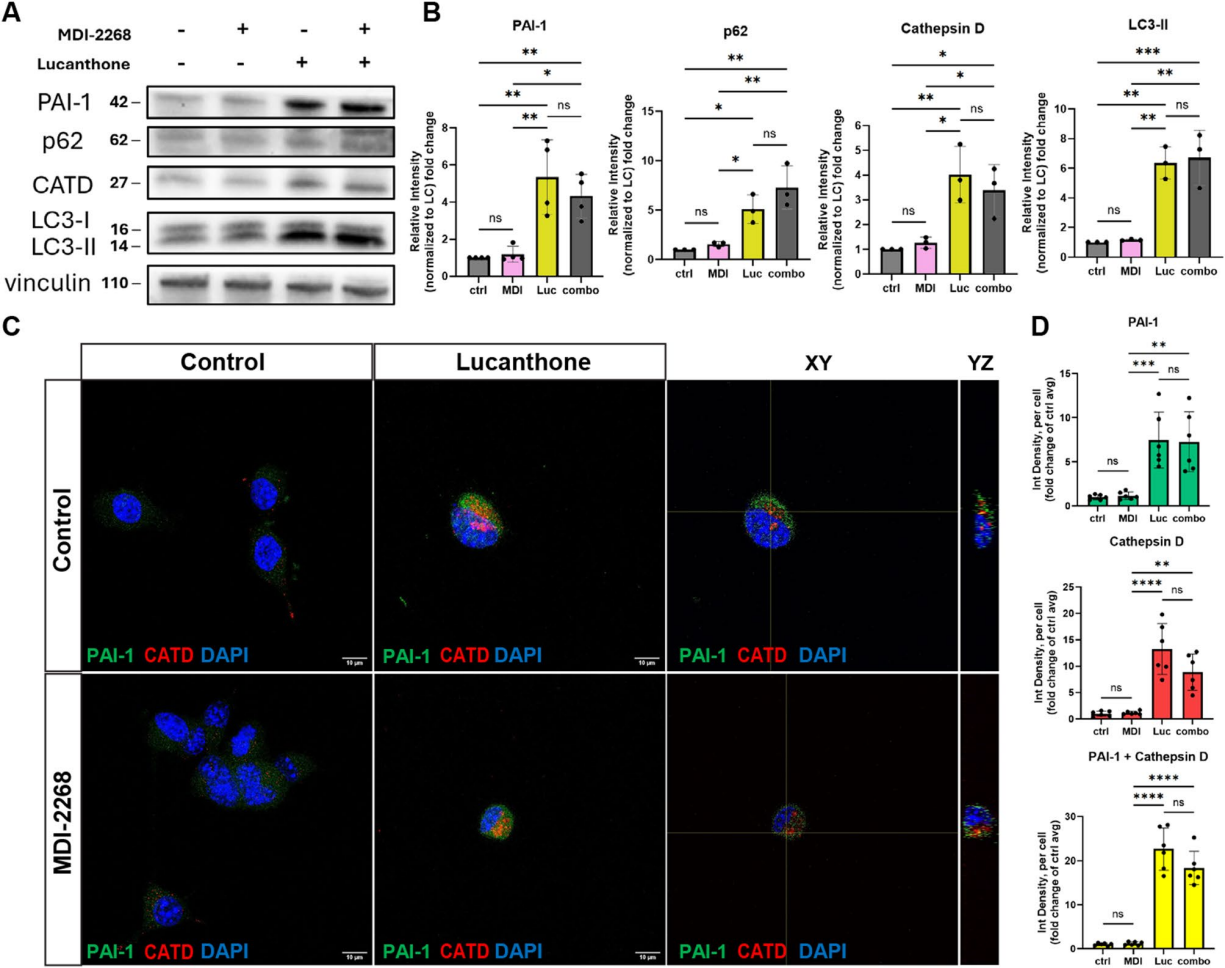
Lucanthone and combination treatments increase autophagy markers and intracellular PAI-1 both within and outside lysosomal machinery. (**A**) Representative western blot of PAI-1, p62, CATD, and LC3 expression in GL261 cells treated with DMSO control, 2.5 µM MDI-2268, 10 µM lucanthone, or the combination, for 24 h. Vinculin was used as loading control. Numbers next to blots are kDa based on protein ladder. (**B**) Quantification of A, normalized to loading control (LC). Data presented as fold change of DMSO control. Each dot represents one independent experiment, one-way ANOVA. (**C**) Representative images and orthogonal views of GL261 cells treated with the above conditions, stained with PAI-1, CATD, and DAPI. Scale bars measure 10 μm. (**D**) Quantification of C. Data presented as fold change of DMSO control. Each dot represents one independent experiment, average of 3–5 images per experiment, one-way ANOVA

Lucanthone and combination with MDI-2268 treatment both markedly increased intracellular PAI-1 and a panel of autophagy proteins, namely, p62, Cathepsin D, and LC3-II; PAI-1 again showed increased colocalization with Cathepsin D (Fig. 4A-D).

To ascertain inhibition of PAI-1 by MDI-2268, the concentration of active PAI-1 in the extracellular space was measured using ELISA assay. Fresh media were removed from cells in culture, and then control (DMSO) or increasing concentrations of MDI-2268 were added directly to the media (Suppl. Figure 5A). The reactions were immediately evaluated by ELISA for PAI-1 activity. Increasing concentrations of MDI-2268 show a concomitant inhibition of PAI-1 activity.

**Fig. 5:**
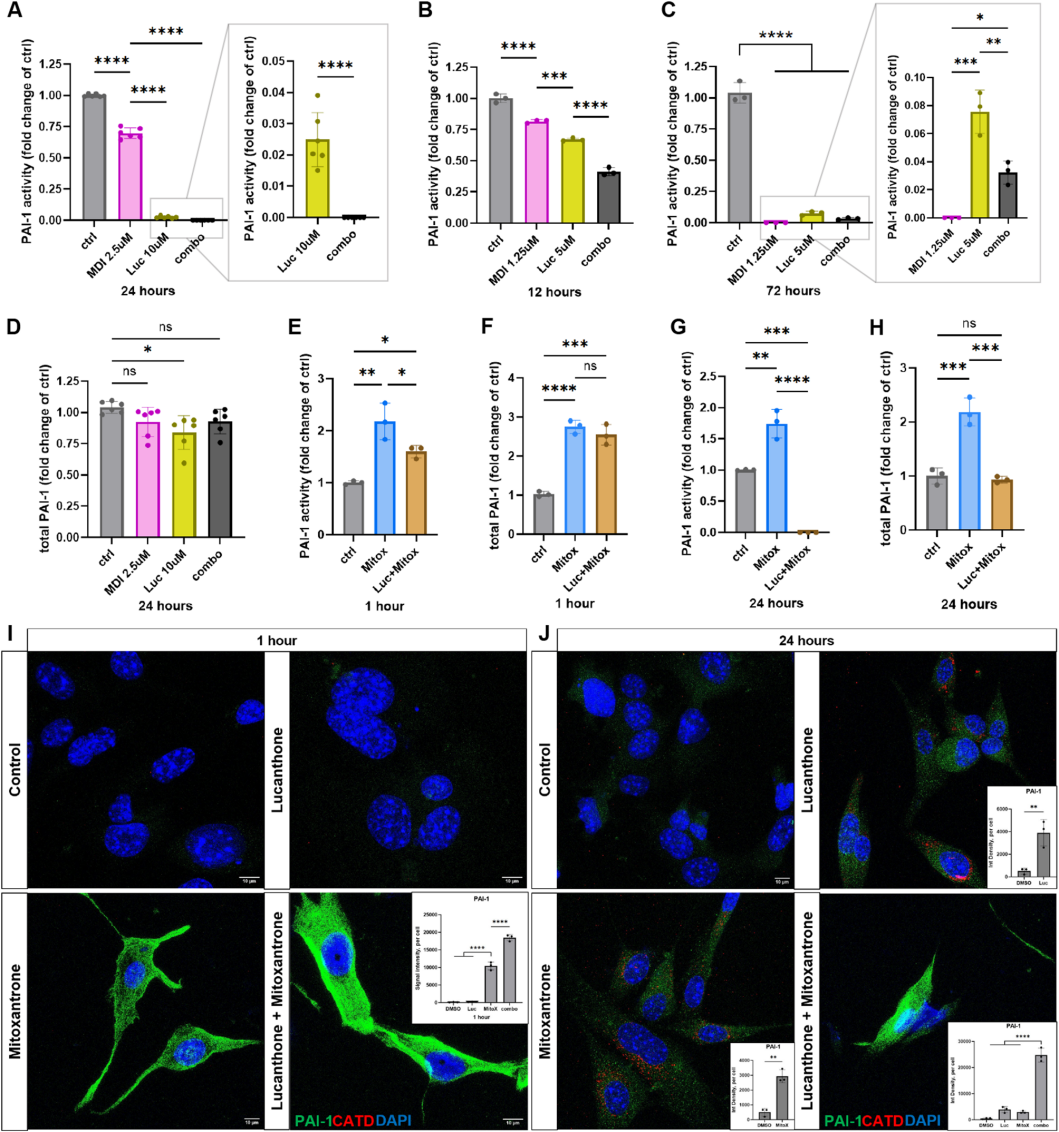
Lucanthone abrogates extracellular active PAI-1. (**A-C**) Active PAI-1 ELISA on conditioned media of GL261 cells treated with DMSO control, either 1.25 or 2.5 µM MDI-2268, 5 or 10 µM lucanthone, or the combination, for 12, 24, or 72 h. Gray boxed plots are zoomed-in views of the y-axis of the depicted conditions. Data presented as fold change of the average of control (DMSO), which was measured in ng/mL of active PAI-1. Each dot represents one independent experiment, one-way ANOVAs, or unpaired t-test for boxed plot in A. (**D**) Total PAI-1 ELISA on culture media of GL261 cells treated with the labeled conditions. Data presented as fold change of the average of control (DMSO). Each dot represents one independent experiment, one-way ANOVA. (**E-H**) Active or total PAI-1 ELISA on conditioned media of GL261 cells treated with DMSO control, 3 µM mitoxantrone, or 3 µM mitoxantrone + 10 µM lucanthone, for 1–24 h. Data presented as fold change of the average of control (DMSO). Each dot represents one independent experiment, one-way ANOVA. (**I, J**) Representative images of GL261 cells treated with control (DMSO), 3 µM mitoxantrone, 10 µM lucanthone, or the combination, at 1 and 24 h, stained with PAI-1, CATD, and DAPI. Quantifications of PAI-1 signal in select conditions shown. Each dot represents one independent experiment, one-way ANOVA in I, one-way ANOVA or unpaired t-tests in J. Scale bars measure 10 μm

Although lucanthone increased intracellular PAI-1 protein levels, both intracellularly and within lysosomes, it significantly reduced active extracellular PAI-1 levels, with no detectable active PAI-1 in the media after 24 h of combination treatment with MDI-2268 (Fig. 5A). Importantly, this occurred without significant change in cell viability (Suppl. Figure 5B), indicating active extracellular PAI-1 reduction was not due to cell death. These effects began to emerge at an earlier time point and lower concentrations, with MDI-2268 inhibiting PAI-1 activity, as expected. Surprisingly, lucanthone further decreased active extracellular PAI-1, and this inhibition of active PAI-1 was exacerbated when the two reagents were combined (Fig. 5B). At the same lower concentrations but 72 h of treatment, MDI-2268 completely abolished PAI-1 activity, lucanthone nearly eliminated it, and the combination provided an intermediate effect (Fig. 5C). Total extracellular PAI-1 protein concentration remained either unchanged or slightly decreased (Fig. 5D). Hence, the vast majority of total secreted PAI-1 in lucanthone and combination-treated cells is inactive. Importantly, since intracellular PAI-1 in these conditions was increased, this result pointed to lucanthone’s potential action in blocking PAI-1 secretion.

To further understand this effect, mitoxantrone, a chemotherapeutic agent known to increase PAI-1 secretion [22] was utilized. Mitoxantrone increased active and total extracellular PAI-1, which, except for total PAI-1 at 1 h post-treatment, was blocked by combining it with lucanthone (Fig. 5E-H, Suppl. Figure 5C, D). This further supports lucanthone’s role in abrogating extracellular PAI-1 activity and secretion. Importantly, this effect does not occur because there is simply less total PAI-1 in the combination, as it was robustly increased intracellularly (Fig. 5I, J). Concomitantly, PAI-1 concentration was either similar to or increased compared to that measured extracellularly in control cells (Fig. 5F, H, Suppl. Figure 5D). Taken together, these results point to the idea that lucanthone can block active PAI-1 secretion and reverse mitoxantrone’s effect of stimulating PAI-1 secretion.

### Combination therapy improved animal survival and reduced tumor volume

**Fig. 6:**
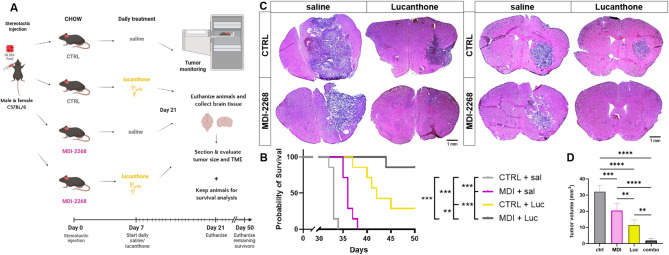
Combination therapy improved animal survival and reduced tumor volume. (**A**) Schematic diagram of in vivo animal study design. (**B**) Kaplan-Meier survival curves for animals receiving control (CTRL chow + saline injections), MDI-2268 (MDI chow + saline injections), Lucanthone (CTRL chow + lucanthone injections), and combination (MDI + lucanthone injections) treatments. 7 animals per group, log-rank (Mantel-Cox) tests. (**C**) Representative images of H&E-stained brain sections for animals in each of the conditions, euthanized at day 21. Comparison of more anterior, bregma 2.25-2.0, (left panel) vs. more posterior, bregma 0.5 − 0.25, (right panel) in the brain. Scale bars measure 1 mm. (**D**) Tumor volume quantification, *n* = 5 animals per condition, one-way ANOVA

To investigate the effects of the two pharmacological agents, lucanthone and MDI-2268, in the GBM model in vivo, they were administered to animals carrying GL261 GBM, as shown in Fig. 6A. Both agents significantly increased median survival time compared to control, with MDI-2268 alone exhibiting a modest, approximately 3-day improvement, while lucanthone treatment had a larger, ~ 9-day, effect (Fig. 6B). However, combined treatment improved survival more than either treatment alone, with nearly all combination animals living to day 50, when all remaining animals were euthanized and their tumors subjected to analysis. This effect was mirrored in histological analyses on animals euthanized on day 21 since tumor cell inoculation: individual drug treatments resulted in decreased tumor volumes, while the combination therapy group yielded a much smaller tumor volume (Fig. 6C, D).

### PAI-1 inhibition enhanced cytotoxic T-cell infiltration, while lucanthone reduced vascularization

**Fig. 7:**
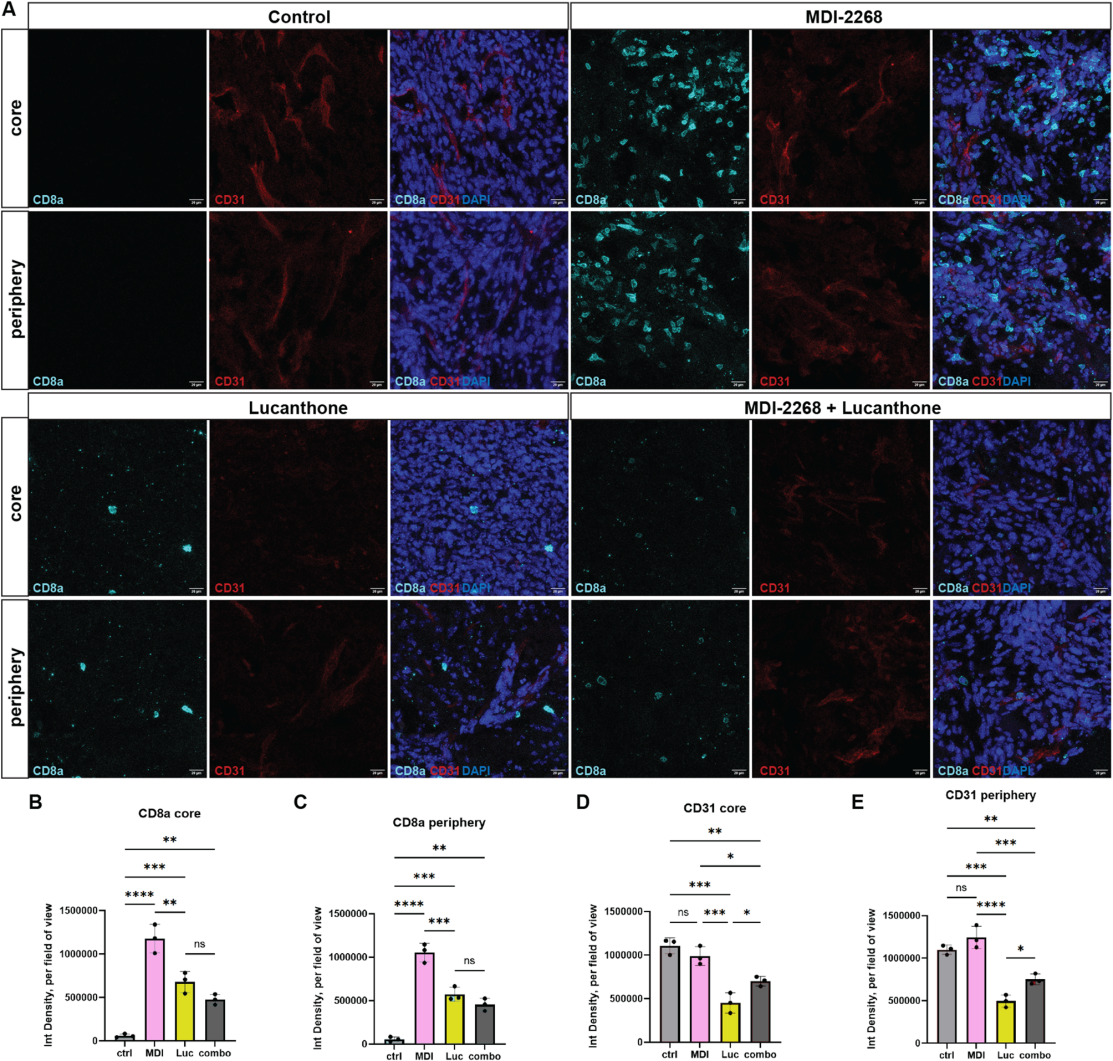
PAI-1 inhibition enhanced cytotoxic T-cell infiltration, while lucanthone reduced vascularization. (**A**) Representative images of tumors at day 21 in the 4 conditions stained with CD8a, CD31, and DAPI. Scale bars measure 20 μm. (**B-E**) Quantifications of A. Each dot represents one animal, average of 3–5 images per animal, one-way ANOVA

To examine potential tumor microenvironmental effects of PAI-1 and autophagy inhibition, immunofluorescence defining specific cell types within the GBM milieu was used. Firstly, although lucanthone- and combination- treated tumors significantly increased CD8a + cytotoxic T-cells, both within the tumor core and at the periphery of the tumor, MDI-2268-treated tumors showed the greatest increase in this cell type (Fig. 7A-C). This result suggests that MDI-2268 may support the development of a successful cancer immune response [45]. On the other hand, when the vascularization in these tumors was assessed, lucanthone treatment was found to significantly reduce CD31 positivity, an endothelial marker of blood vessels [46]both inside and at the border of the tumor (Fig. 7A-E), a finding consistent with our previous work [8]. PAI-1 inhibition, which has previously been shown to both increase and decrease angiogenesis depending on the context [19, 47]did not significantly change CD31 + vascularization, and interestingly, combination therapy showed an intermediate effect, partially rescuing the lucanthone-mediated CD31 decrease.

### MDI-2268 and lucanthone treatments decreased Arginase 1 and CD206 expression in vivo

**Fig. 8:**
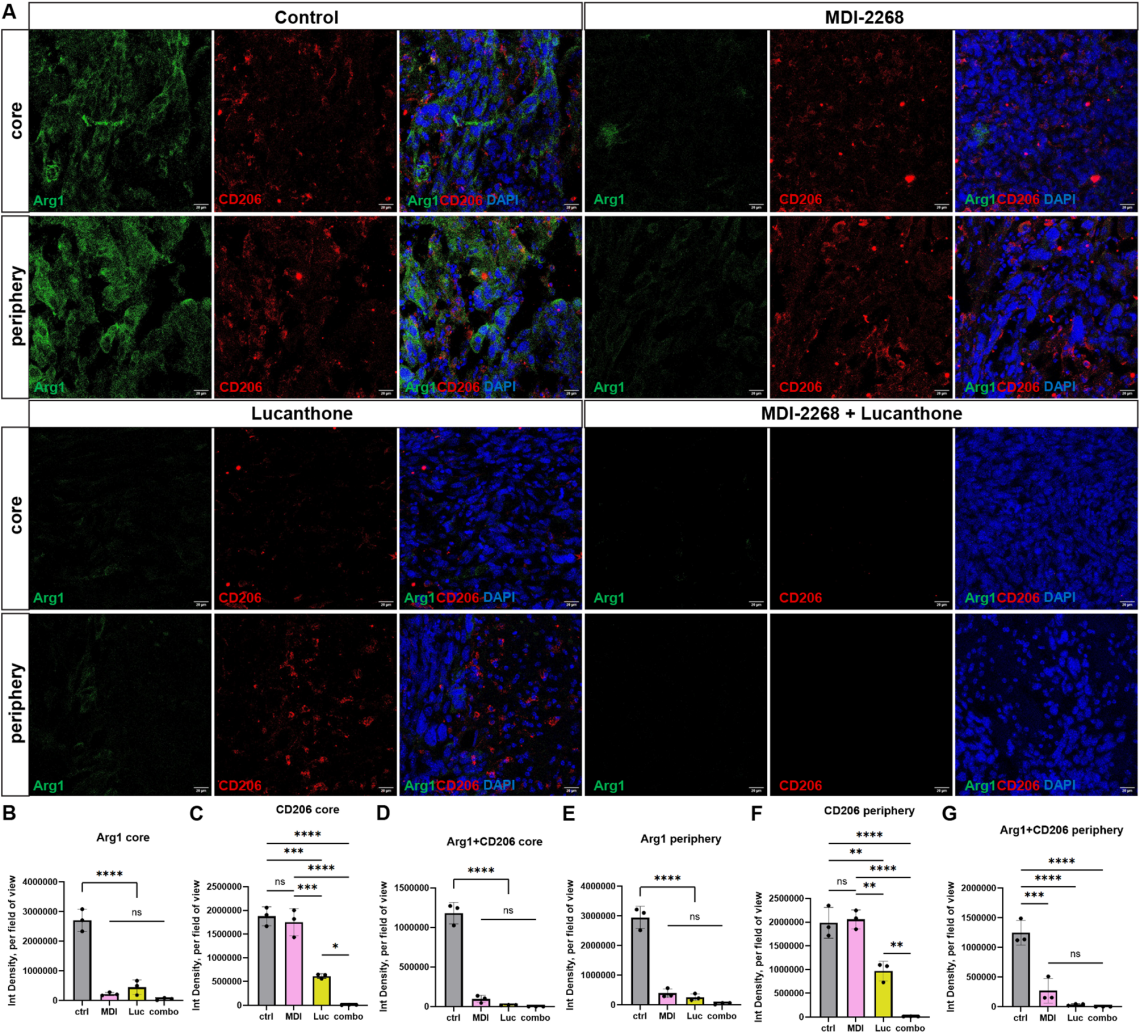
MDI-2268 and lucanthone treatments decreased Arginase 1 and CD206 expression in vivo. (**A**) Representative images of tumors at day 21 in the 4 conditions stained with Arginase 1, CD206, and DAPI. Scale bars measure 20 μm. (**B-G**) Quantifications of A. Each dot represents one animal, average of 3–5 images per animal, one-way ANOVA

Myeloid cells are an essential part of the innate immune system and can express anti- or pro- inflammatory genes and secrete corresponding factors, thus polarizing towards suppressing or stimulating other effector immune cells [11]. Myeloid cells found in the brain are brain-resident microglia and peripherally derived macrophages, or monocytes [48]. To investigate immune-suppressive tumor signature in response to these therapies, expression of anti-inflammatory markers Arginase1 (Arg1) and CD206 were assessed (Fig. 8A). Arg1 expression was dramatically decreased in MDI-2268, lucanthone, and combination treated tumors, within the tumor core and at the periphery (Fig. 8B, E). Interestingly, PAI-1 inhibition alone did not significantly alter CD206 expression, but autophagy inhibition alone did significantly decrease CD206 expression, notably, more so within tumor cores than outside tumor borders (Fig. 8C, F). In combination treated tumors, both Arg1 and CD206 expression were barely detectable. Moreover, Arg1 and CD206 colocalized the most within control-treated tumors, and this colocalization was substantially reduced in all other treatment groups (Fig. 8D, G). By contrast, IL-1β, a pro-inflammatory secreted factor [11] was increased in lucanthone and combination treated tumors (Suppl. Figure 6), supporting the notion that these treatments may have polarized tumors to a more immune-stimulatory composition.

### Inhibition of both autophagy and PAI-1 activated immune-stimulatory myeloid cells

**Fig. 9:**
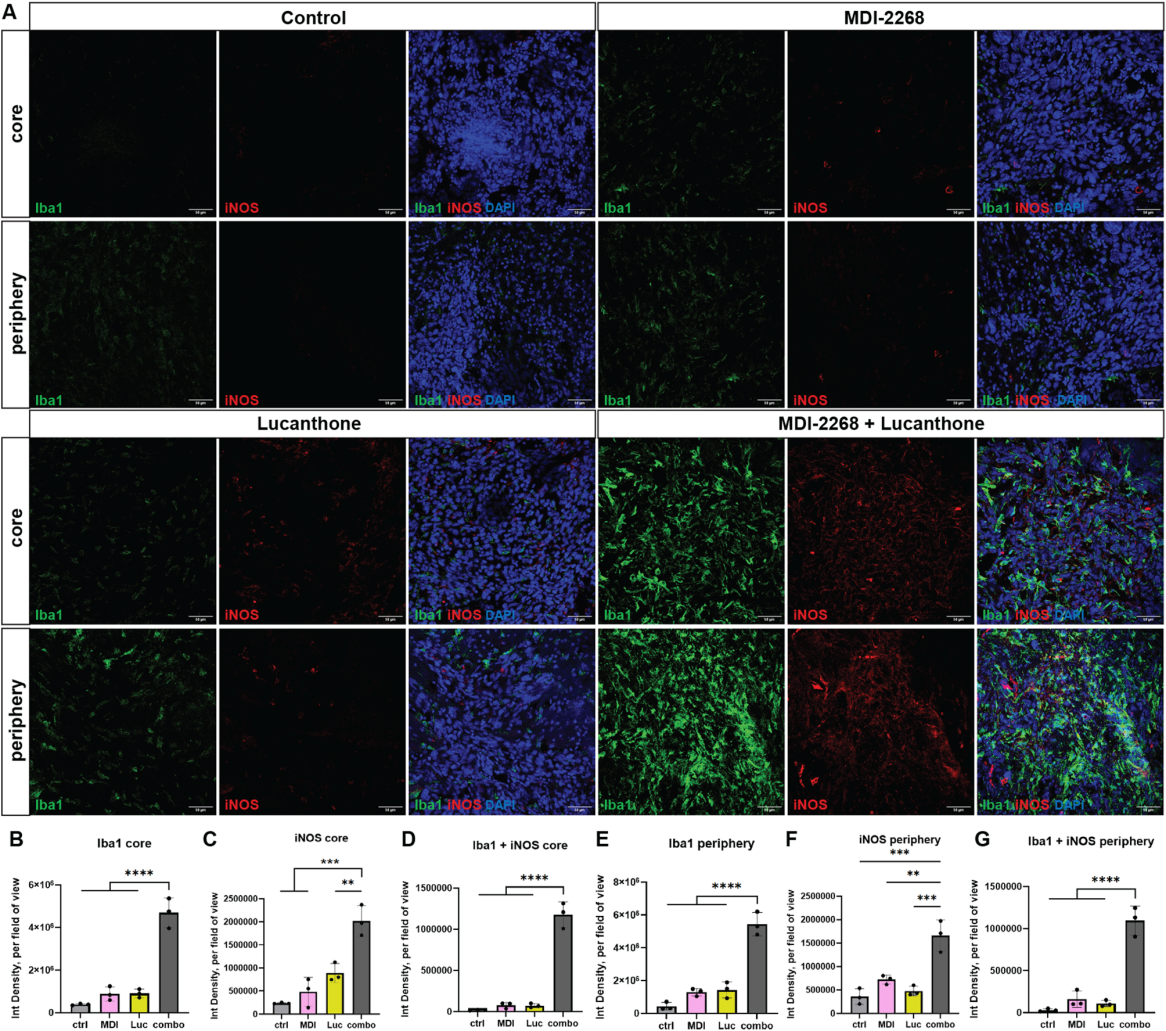
Inhibition of both autophagy and PAI-1 activated immune-stimulatory myeloid cells. (**A**) Representative images of tumors at day 21 in the 4 conditions stained with Iba1, iNOS, and DAPI. Scale bars measure 50 μm. (**B-G**) Quantifications of A. Each dot represents one animal, average of 3–5 images per animal, one-way ANOVA

Finally, to elucidate immune-stimulatory tumor phenotypes, expression of Iba1, specific to activated microglia and macrophages, and iNOS, a pro-inflammatory marker for microglia and macrophages, were evaluated. Our studies revealed that the combination of PAI-1 and autophagy inhibition dramatically increased infiltration and activation of microglia and macrophages (Iba1+) in the tumor, polarized to an immune-stimulatory signature (iNOS+) (Fig. 9A-G). To validate these myeloid cell responses, mouse microglia cell lines were utilized in conditioned media (CM) experiments. Iba1 expression was increased in microglia that had been incubated with PAI-1-depleted glioma CM (Suppl. Figure 7 A, B). When microglia were incubated with CM from glioma cells treated with the same conditions described in prior in vitro experiments, iNOS and Iba1 expression were significantly increased in lucanthone and combination CM microglia (Suppl. Figure 7C, D). Taken together, our findings suggest a powerful innate immune cell-mediated antitumor effect through inhibition of PAI-1 activity in the TME.

## Discussion

In this study, we demonstrate that glioblastoma (GBM) tumors upregulate PAI-1 in response to autophagy inhibition by lucanthone, and that PAI-1 plays a critical role in tumor survival and immune modulation. We show that PAI-1 localizes to autophagy-related vesicles and that lucanthone increases intracellular PAI-1 while reducing its active extracellular form. Pharmacological or genetic inhibition of PAI-1 reduces glioma cell viability, stemness, and invasion, and enhances immune-stimulatory features in the tumor microenvironment. Notably, combining lucanthone with the PAI-1 inhibitor MDI-2268 synergistically reduces tumor burden, prolongs survival, and promotes a shift toward a pro-inflammatory immune landscape.

These findings highlight PAI-1 as a significant marker for GBM prognosis, particularly pronounced within the proneural subtype, and identify PAI-1 as a GBM-intrinsic response to autophagy inhibition by lucanthone. Morphologically, both higher (Fig. 1F) and lower (Suppl. Figure 7) magnification views showed that lucanthone-treated tumors exhibited cells that appeared enlarged, with increased nuclear and cytoplasm volume compared to cells within control-treated tumors. This is consistent with autophagy blockade by lucanthone and accumulation of undegraded proteins and cell swelling, as has been observed by our previous studies [8, 9]. Our data also emphasize that combined inhibition of autophagy and PAI-1 produced additive effects to decrease glioma cell viability and invasiveness, and enhanced animal survival through distinct, but complementary mechanisms.

Mechanistically, lucanthone increased intracellular PAI-1 while reducing its active extracellular form, suggesting a disruption in PAI-1 secretion or activation. Although the precise mechanism remains unclear, our data suggest that lucanthone may impair secretory autophagy, leading to intracellular retention of PAI-1. This is supported by the observation that lucanthone-treated cells showed strong colocalization of PAI-1 with lysosomal markers (LAMP1, Cathepsin D) and Rab7a, but not with Rab5 or Rab27 isoforms, which are associated with early endosomes and exosomes, respectively. A limitation of this study is the inability to directly measure intracellular PAI-1 activity, as PAI-1 is known to rapidly convert into its latent form [49]. Since extracellular PAI-1 is free in the conditioned media, it can be easily evaluated by active PAI-1 ELISA, whereas examining intracellular protein activity by the same method requires cell lysis. When cell lysates were evaluated, PAI-1 activity was not detected (data not shown). It is possible that this is due to the lysis buffer, which may denature proteins and might impact PAI-1 activity; thus, subsequent work could investigate how to optimize the conditions for preserving protein activity. It may also be feasible to detect the fraction of PAI-1 complexed to plasminogen activators vs. non-complexed, or latent, PAI-1 by immunoblot [50]. On the other hand, employing methods such as proximity ligation assay to tPA [51] or testing a PAI-1 active site-specific antibody for immunofluorescence [52] could illuminate PAI-1 activity and localization on a subcellular level.

Interestingly, some MDI-2268/lucanthone combination treatment effects were intermediate rather than additive: namely, in PAI-1 activity with lower concentrations, in Olig2 protein levels in GSC, and in tumor vascularization. This may reflect a complex interplay between PAI-1 expression, secretion, and inhibition, where lucanthone-induced PAI-1 upregulation may partially counteract MDI-2268’s inhibitory effects. These dynamics, of a heterogenous GBM tumor responding to these treatments through active and/or latent PAI-1, warrant further investigation to optimize dosing and scheduling strategies.

PAI-1 intracellular localization and secretion have been studied in different settings, most abundantly in platelets, as these cells store and release PAI-1 in response to hemodynamic changes [53–55]. PAI-1 is stored primarily in alpha granules of platelets and is reported to be released by the regulated secretory pathway [53, 54]; however, further work showed that PAI-1 activity is stabilized by acidic pH and found within lysosomes [55, 56]. Since autophagy inhibitors, including lucanthone, reduce the acidity of lysosomes [8, 9, 57] this could explain why the vast majority of extracellular PAI-1 is inactive with lucanthone treatment.

PAI-1 cellular localization has been studied in other cell types and shown to localize both within the cytoplasm and the nucleus [58–60]. Nonetheless, the location of PAI-1 within mammalian cells, especially in glioma, has remained mostly unexplored. As part of this study, we demonstrate that PAI-1 colocalizes with autophagy marker Cathepsin D after blockade of this cellular process by lucanthone, thus supporting the notion that PAI-1 is involved in and secreted by autophagy in GBM. However, as PAI-1 is also abundant outside of Cathepsin D + areas, we attempted to localize intracellular PAI-1 in the glioma model after lucanthone stimulation. Although PAI-1 colocalized more with autophagy-related markers LAMP1 and Rab7a, there was still abundant PAI-1 signal outside the lysosomal markers (Suppl. Figure 3). Thus, it remains unclear exactly which cellular vesicular components PAI-1 is part of in glioma cells. It is possible that when stimulated by lucanthone, PAI-1 is both free-floating within the cytoplasm and within a different type of cytoplasmic vesicle.

Intriguingly, PAI-1 inhibition alone resulted in the greatest increase in CD8 + T-cell infiltration within tumors, suggesting a role of PAI-1 in immune evasion. The increase in CD8 + cells with MDI-2268 alone is in agreement with a recent study investigating PAI-1, PD-L1 and CD8 + cells in various tumor types [24]. Part of that work shows PAI-1 inhibition resulted in an increase in CD8 positivity in tumors, and that addition of PAI-1 ablated CD8 + T-cell activation. A different study in melanoma also reported that PAI-1 inhibition led to an increase in CD8 + cells within the tumor [61]. The finding that lucanthone treatment results in increased PAI-1 could explain why the lucanthone- and combination-treated tumors show lower CD8 + T-cells compared to MDI-2268 treated tumors. Additionally, it is possible that lucanthone, while slowing tumor growth, may also directly affect T-cells, which could be another reason for the reduction in T-cells seen in lucanthone conditions compared to PAI-1-inhibited tumors. Nevertheless, the findings in this study corroborate and extend our previous work, which also showed increased T-cell infiltration with lucanthone compared to control treatment [8]. Finally, the robust T-cell presence in MDI-2268-treated tumors could function as the driver of the tumor volume decrease and animal survival time increase compared to control.

Our findings unveiled a vast reduction in Arginase 1, a marker of immune suppression, in MDI-2268, lucanthone, and combination treatment groups compared to control. CD206, expressed by myeloid immune cells exhibiting an anti-inflammatory polarization, was also decreased in lucanthone and combination groups, and combination-treated tumors had nearly undetectable signal for Arg1, CD206, or their colocalization. In murine and human gliomas, both malignant cells and tumor-infiltrating myeloid cells have been found to upregulate the expression of Arg1, and the resulting changes in L-arginine metabolism are a critical mechanism contributing to immune suppression [62]. On the other hand, CD206 is known to be a marker specific to immuno suppressive myeloid cells [63]. Thus, it is possible that PAI-1 inhibition alone did not change the amount of infiltrating immuno suppressive microglia and macrophages (CD206+) but did decrease Arg1 expression throughout the tumor. The combination treatment and reduction of immunosuppressive markers such as Arginase 1 and CD206 indicated a shift toward a more immune-permissive tumor microenvironment. We also observed activation of microglia and macrophages in response to combination treatment, as evidenced by increased Iba1 and iNOS expression. Conditioned media experiments confirmed that PAI-1-depleted or lucanthone- and combination-treated glioma cells promote a pro-inflammatory phenotype in microglia, further supporting the immunomodulatory potential of this therapeutic strategy.

**Fig. 10:**
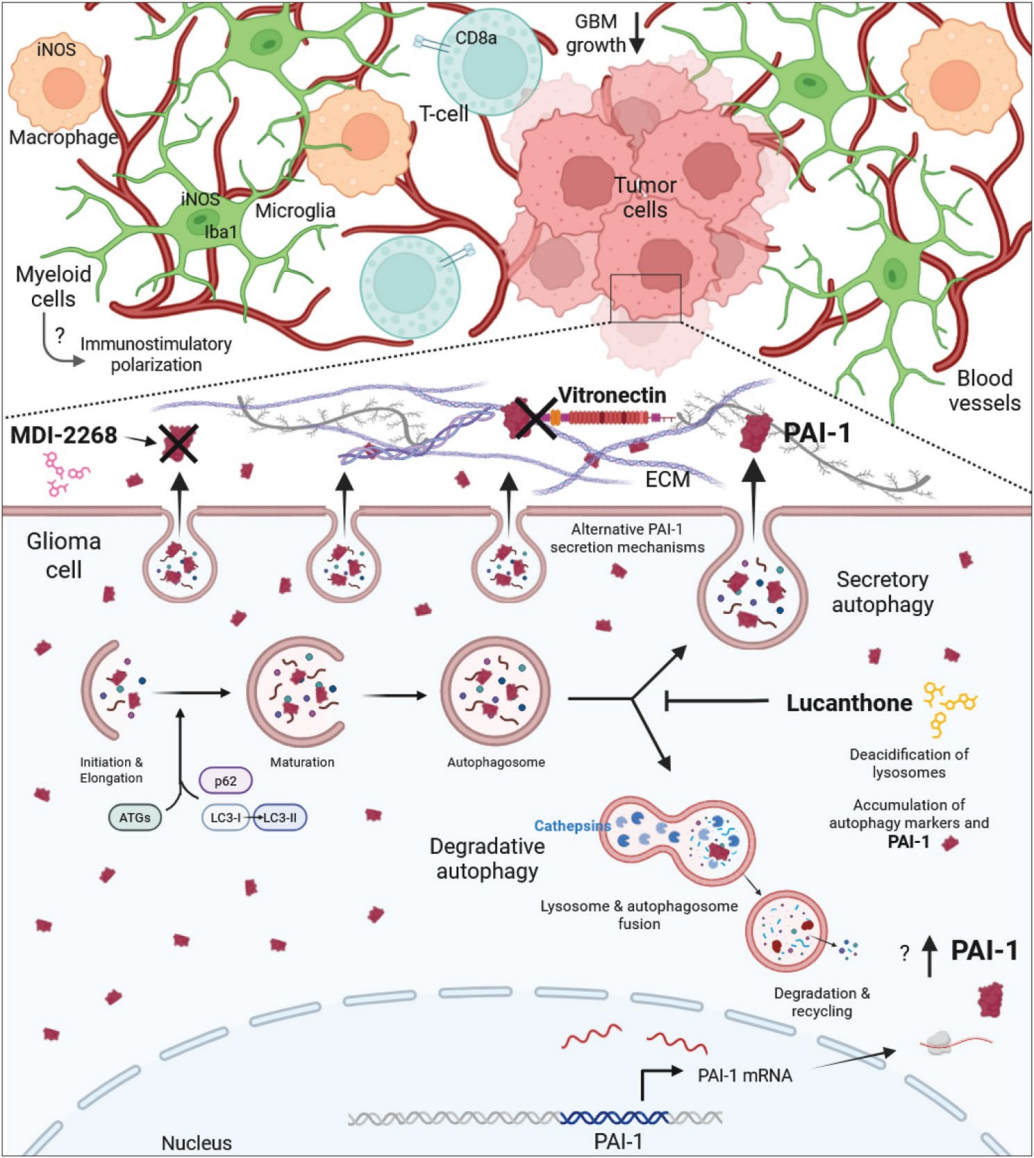
Schematic diagram of the proposed role of PAI-1 in lucanthone treatment response and how the combination of PAI-1 and autophagy inhibition may act on the intra- and extra-cellular level in GBM. The bottom half of the image depicts a zoomed-in view of the boxed area of a glioma cell in the top portion, challenged with MDI-2268 and lucanthone, and the top depicts a complex TME (not to scale), with labeled tumor cells in light red, blood vessels in dark red, cytotoxic T-cells in blue, microglia in green, and peripheral macrophages in orange, responding to this treatment. Lucanthone increases PAI-1 intracellularly, results in a buildup of undegraded proteins, including PAI-1, inside deacidified lysosomes, and blocks PAI-1 in the extracellular space. Thus, lucanthone is proposed to inhibit both degradative and secretory autophagy. MDI-2268 inhibits PAI-1’s activity and vitronectin binding in the extracellular matrix (ECM), and its combination with lucanthone results in a dramatic reduction in GBM growth, prolonged animal survival, and an immunostimulatory myeloid cell response. Question marks depict mechanisms that remain to be elucidated, i.e. precisely how lucanthone leads to increased PAI-1 outside autophagic machinery, and the method of activation of microglia and macrophages. This figure was created in Biorender

The current working model of the role of PAI-1 in the tumoral response to PAI-1 and autophagy inhibition in GBM is depicted in Fig. 10. Together, these results suggest that dual targeting of autophagy and PAI-1 both impairs tumor cell survival and potentially reprograms the tumor microenvironment to favor anti-tumor immunity. The immune landscape of the PAI-1 and autophagy inhibited tumors appears more favorable towards myeloid cell activation, resulting in decreased tumor volume and longer animal survival. Future mechanistic studies should explore the broader impact of this combination on secretory autophagy and immune signaling, and assess its translational potential in human GBM models.

## Conclusions and future directions

Our study identifies PAI-1 as a critical mediator of glioblastoma (GBM) resistance to autophagy inhibition and highlights its dual role in tumor cell survival and immune modulation. We demonstrate that lucanthone, a brain-penetrant autophagy inhibitor, induces intracellular accumulation of PAI-1 while reducing its active extracellular form. This effect is associated with impaired secretory autophagy and a shift in the tumor microenvironment toward a more immunο-stimulatory state. Importantly, combining lucanthone with the selective PAI-1 inhibitor MDI-2268 synergistically suppressed tumor growth, prolonged survival, and enhanced immune activation in vivo. These findings support a potential therapeutic strategy targeting both autophagy and PAI-1 to disrupt tumor-intrinsic survival mechanisms and reprogram the tumor microenvironment. However, several mechanistic questions remain. Future studies should aim to elucidate the precise pathways by which lucanthone impairs PAI-1 secretion, determine the activity state of intracellular PAI-1, and assess whether other autophagy-dependent secreted factors [64] are similarly affected. Additionally, further investigation is needed to define the cellular sources and functional roles of immune cell subsets modulated by this combination therapy. Expanding this work into human GBM models and patient-derived systems will be essential to evaluate translational potential and therapeutic efficacy.

Overall, our results provide compelling evidence that dual inhibition of autophagy and PAI-1 represents a promising approach to overcome GBM resistance and stimulate anti-tumor immunity.

## Electronic supplementary material

Below is the link to the electronic supplementary material.

Supplementary Material 1

Supplementary Material 2

Supplementary Material 3

Supplementary Material 4

Supplementary Material 5

Supplementary Material 6

Supplementary Material 7

Supplementary Material 8

Supplementary Material 9

Supplementary Material 10

## Data Availability

No datasets were generated or analysed during the current study.

## Abbreviations

GBM: Glioblastoma
PAI-1: Plasminogen activator inhibitor-1
SERPINE1: Serine protease inhibitor family E member 1
TME: Tumor microenvironment
PFA: Paraformaldehyde
CATD/CTSD: Cathepsin D
p62/SQSTM1: Sequestosome-1
LC3: Microtubule-associated protein 1 A/1B-light chain 3
LAMP1: Lysosomal-associated membrane protein 1
