# Supplementary material for: Targeting autophagy and plasminogen activator inhibitor-1 increases survival and remodels the tumor microenvironment in glioblastoma
Source: J Exp Clin Cancer Res. 2025 Jul 19;44:214. doi: 10.1186/s13046-025-03473-w (PMC12275254; doi:10.1186/s13046-025-03473-w)
Supplement: Supplementary file 1 — Supplementary Material 1 [file 13046_2025_3473_MOESM1_ESM.docx]

**Supplementary Table 1.** List of primary antibodies used.

| **Antibody** | **Application** | **Dilution** | **Company, Catalog #** |
| --- | --- | --- | --- |
| PAI-1 | ICC, IHC | 1:500 | Novus Bio, NBP1-19773 |
| PAI-1 | WB | 1:1000 | CST, 27535S |
| PAI-1 | WB | 1:1000 | Innov Res, ISHAHUPAI1AP100UG |
| Cathepsin D | WB, ICC, IHC | 1-10 ug/mL | R&D Systems, AF1029 |
| Ki67 | ICC | 1:250 | Thermo Fisher, 14-5698-82 |
| Cleaved Caspase 3 | ICC | 1:250 | CST, 9664S |
| LAMP1 | ICC | 1:250 | Santa Cruz, sc-19992 |
| CD8a | IHC | 1:250 | Abcam, ab217344 |
| CD31 | IHC | 1:250 | BD Biosciences, 557355 |
| Iba1 | IHC, WB | 1:500 | Wako, 019-19741 |
| iNOS | IHC, WB | 1:500 | BD Biosciences, 610431 |
| LC3 | WB | 1:1000 | CST, 2775S |
| p62 | WB | 1:500 | Novus Bio, NBP1-48320 |
| Arginase 1 | IHC | 5 ug/mL | BD Biosciences, 610708 |
| CD206 | IHC | 5 ug/mL | R&D Systems, AF2535 |
| IL-1β | IHC | 5 ug/mL | R&D Systems, AF-401-SP |
| Olig2 | WB | 5 ug/mL | R&D Systems, AF2418 |
| CD86 | WB | 1:500 | BD Biosciences, 553689 |
| Nestin | WB | 1:500 | Abcam, ab6142 |
| S100B | WB | 1:500 | Proteintech, 15146-1-AP |
| Vinculin | WB | 1:1000 | Santa Cruz, sc-73264 |
| GAPDH | WB | 1:2000 | Santa Cruz, sc-32233 |
| β-Actin | WB | 1:2000 | Sigma Aldrich, A2228 |
