# Supplementary material for: Targeting autophagy and plasminogen activator inhibitor-1 increases survival and remodels the tumor microenvironment in glioblastoma
Source: J Exp Clin Cancer Res. 2025 Jul 19;44:214. doi: 10.1186/s13046-025-03473-w (PMC12275254; doi:10.1186/s13046-025-03473-w)
Supplement: Supplementary file 3 — Supplementary Material 3 [file 13046_2025_3473_MOESM3_ESM.docx]

**Supplementary Figure Legends**

**Supplementary Figure 1. PAI-1 and Cathepsin D are increased in tumors treated with lucanthone.** Lower magnification representative confocal images of tumors treated with saline control or lucanthone for two weeks and stained with PAI-1, Cathepsin D (CATD), and DAPI nuclear stain. Scale bars measure 10 µm.

**Supplementary Figure 2. Lucanthone increases relative PAI-1 expression and protein level. A.** Relative mRNA expression evaluated by q-RT-PCR of PAI-1 in GL261 cells treated with DMSO (control) or 10 µM lucanthone for 24 hours, normalized to GAPDH. Data presented as fold change of the average of control. Each dot represents one independent experiment, average of 3 technical replicates, unpaired t-test. **B.** Western blot of PAI-1 expression in patient-derived glioma cell lines, GBM9 and GBM43, treated with DMSO control or 5 µM lucanthone for 24 hours. GAPDH was used as loading control.

**Supplementary Figure 3. PAI-1 colocalizes with Rab7a and LAMP1 more than it does with Rab5, Rab27a or Rab27b. A-E.** Representative images of GL261 cells transfected with Rab7a, Rab5, Rab27a, or Rab27b-GFP (magenta), treated with 10 µM lucanthone for 24 hours, and then stained with PAI-1 (green), LAMP1 (red), and DAPI (blue). All scale bars measure 10 µm. **A.** PAI-1 is shown in green (top and bottom), Rab7a is shown in magenta (top) and LAMP1 is shown in red (bottom) of the same field of view. **B.** Lower magnification images of merged PAI-1/Rab7a/DAPI (top), and merged PAI-1/LAMP1/DAPI (bottom) of the same field of view. **C.** Merged PAI-1/Rab5/DAPI and PAI-1/LAMP1/DAPI of the same field of view. **D.** Merged PAI-1/Rab27a/DAPI and PAI-1/LAMP1/DAPI of the same field of view. **E.** Merged PAI-1/Rab27b/DAPI and PAI-1/LAMP1/DAPI of the same field of view. **F.** Quantification of the colocalization of PAI-1 integrated density with each of the Rab-GFP constructs (7a, 5, 27a, 27b) in comparison to the colocalization of PAI-1 with LAMP1 in the same 4 transfection conditions, divided by the number of Rab-positive cells per field of view. Each shape represents one independent experiment, average of 3-5 images per experiment, two-way ANOVA.

**Supplementary Figure 4. PAI-1 knockdown and inhibition decreases glioma cell viability. A, B.** Two mouse glioma cell lines, GL261 and KR158, were incubated with 1.25 µg/mL control siRNA LNPs or 1.25 µg/mL siRNA targeting mouse PAI-1 LNPs for 72 hours, PAI-1 knockdown was confirmed by western blot with GAPDH used as loading control, and cell viability (% live cell count relative to average of control) was measured by MTT assay. n=3 independent experiments, unpaired t-tests. **C.** MTT assays measuring cell viability of GL261 cells incubated with increasing concentrations (in µM) of either MDI-2268 or lucanthone for 72 hours. Each dot and error bar represent mean +/- SD for 3 independent experiments per condition. **D.** Representative western blot of Nestin, Olig2, S100B, and Iba1 expression in glioma stem cell (GSC) or adherent (adh) GL261 cells that were treated with DMSO control, 2.5 µM MDI-2268, 10µM lucanthone, or the combination, for 24 hours. β-actin was used as loading control. **E-G.** Quantifications of E, normalized to loading control (LC). Data presented as fold change of DMSO control. Each dot represents one independent experiment, one-way ANOVAs. **H.** Representative images taken at 10x magnification of invasion assay of GL261 cells treated with DMSO control, 2.5 µM MDI-2268, 10µM lucanthone, or the combination, for 24 hours, and stained with Crystal Violet. Quantification of invasion assay. Data presented as % invaded cells relative to DMSO control. Each dot represents one independent experiment, one-way ANOVA.

**Supplementary Figure 5. MDI-2268 reduces PAI-1 activity and lucanthone abrogates extracellular active PAI-1. A.** Fresh media was taken from GL261 cells in culture and DMSO or increasing concentrations of MDI-2268 (in µM) was added directly to the media, and immediately evaluated by ELISA for PAI-1 activity. Data presented as fold change of the average of control (DMSO). Each dot represents one independent experiment, one-way ANOVA. **B.** MTT assay measuring GL261 cell viability at 24 hours of the labeled conditions, n=5 independent experiments, one-way ANOVA. **C. D.** Active or total PAI-1 ELISA on conditioned media of GL261 cells treated with DMSO control, 3 µM mitoxantrone, or 3 µM mitoxantrone + 10 µM lucanthone, for 72 hours. Data presented as fold change of the average of control (DMSO). Each dot represents one independent experiment, one-way ANOVA, except for ctrl and Luc+Mitox comparisons, where t-tests were used.

**Supplementary Figure 6.** **Lucanthone and combination treatments increased IL-1β expression in vivo. A.** Representative images of tumors at day 21 in the 4 conditions stained with Arginase 1, IL-1β, and DAPI. Scale bars measure 20 µm. **B-G.** Quantifications of A. Each dot represents one animal, average of 3-5 images per animal, one-way ANOVA.

**Supplementary Figure 7. PAI-1-depleted and -inhibited glioma conditioned media increased pro-inflammatory markers in microglia, while PAI-1 and autophagy inhibition reduced glioma stem-like markers and invasion. A.** Representative western blot of iNOS, CD86, and Iba1 expression in BV2 mouse microglia cells incubated with conditioned media for 24 hours of GL261 cells that were treated with either control siRNA LNPs (siCtrl) or siRNA targeting mouse PAI-1 LNPs (siPAI-1) for 72 hours. β-actin was used as loading control. **B.** Quantifications of A, normalized to loading control (LC). Data presented as fold change of siCtrl. Each dot represents one independent experiment, unpaired t-tests. **C.** Representative western blot of iNOS, CD86, and Iba1 expression in N9 mouse microglia cells incubated with conditioned media for 24 hours of GL261 cells that were treated with DMSO control, 2.5 µM MDI-2268, 10µM lucanthone, or the combination, for 24 hours. β-actin was used as loading control. **D.** Quantifications of C, normalized to loading control (LC). Data presented as fold change of DMSO control. Each dot represents one independent experiment, one-way ANOVAs.
