# Supplementary figures and images for: Targeting autophagy and plasminogen activator inhibitor-1 increases survival and remodels the tumor microenvironment in glioblastoma
Source: J Exp Clin Cancer Res. 2025 Jul 19;44:214. doi: 10.1186/s13046-025-03473-w (PMC12275254; doi:10.1186/s13046-025-03473-w)

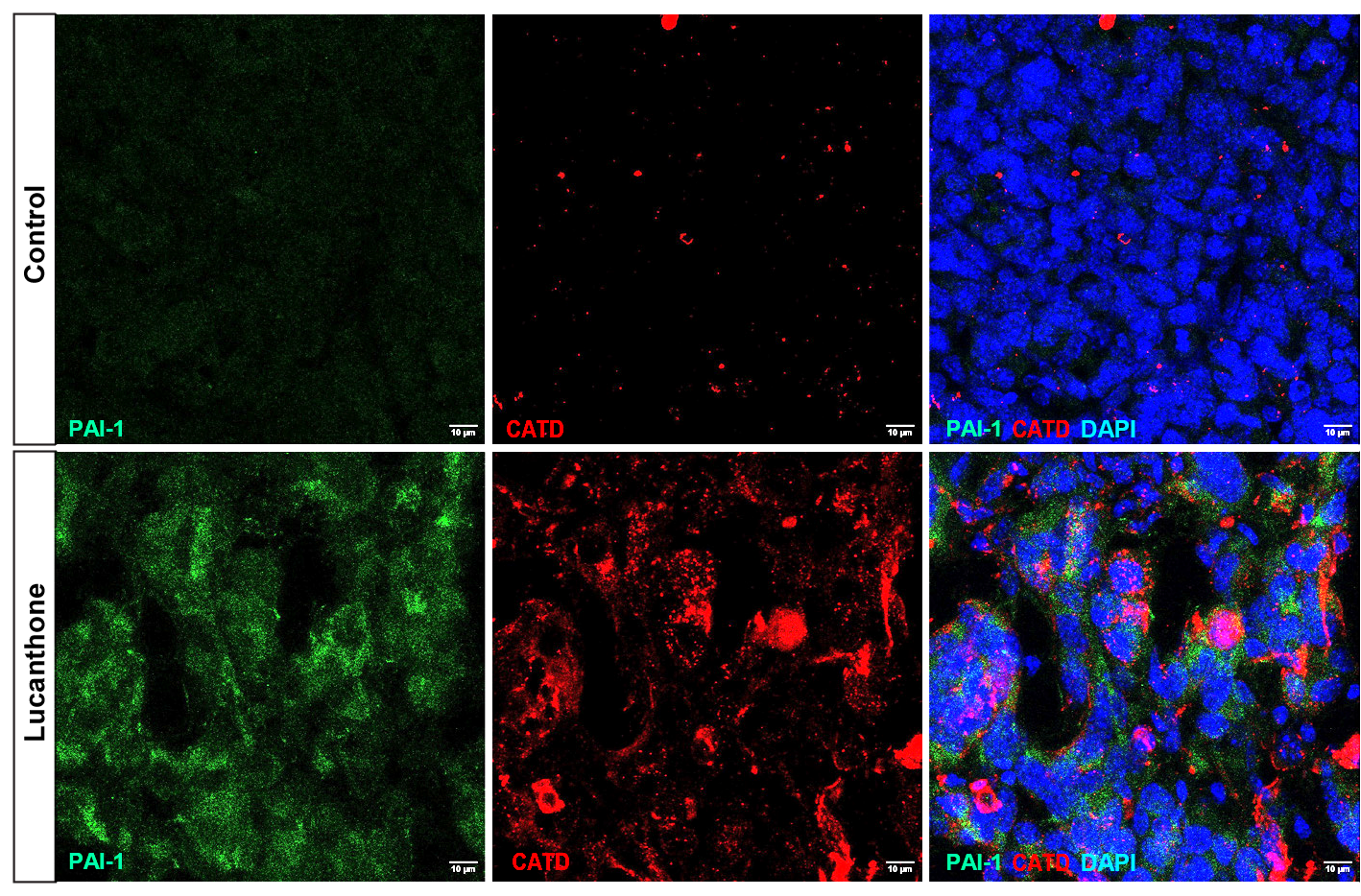

Supplement: Supplementary file 4 — Supplementary Material 4 [file 13046_2025_3473_MOESM4_ESM.tif]

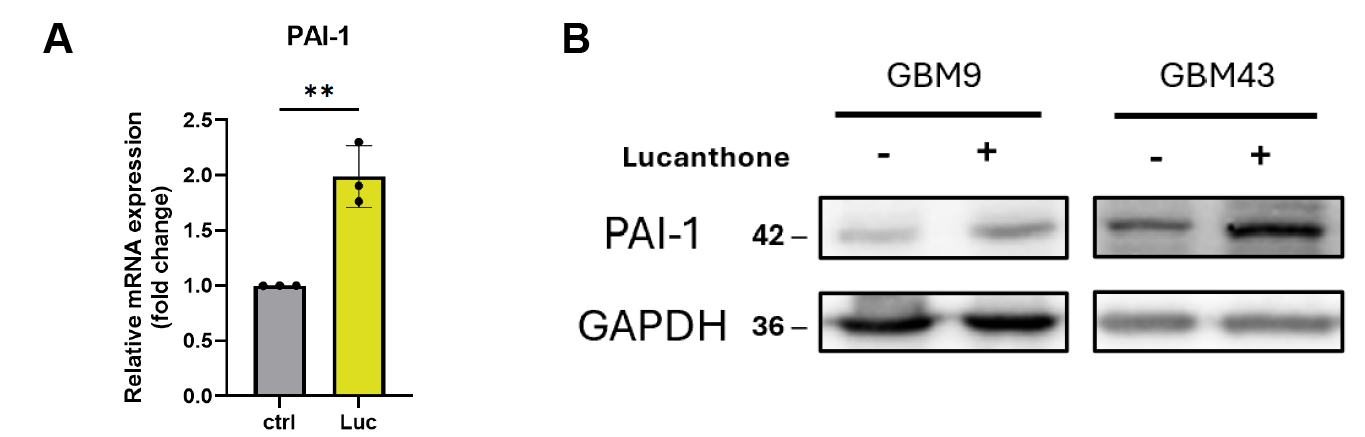

Supplement: Supplementary file 5 — Supplementary Material 5 [file 13046_2025_3473_MOESM5_ESM.tif]

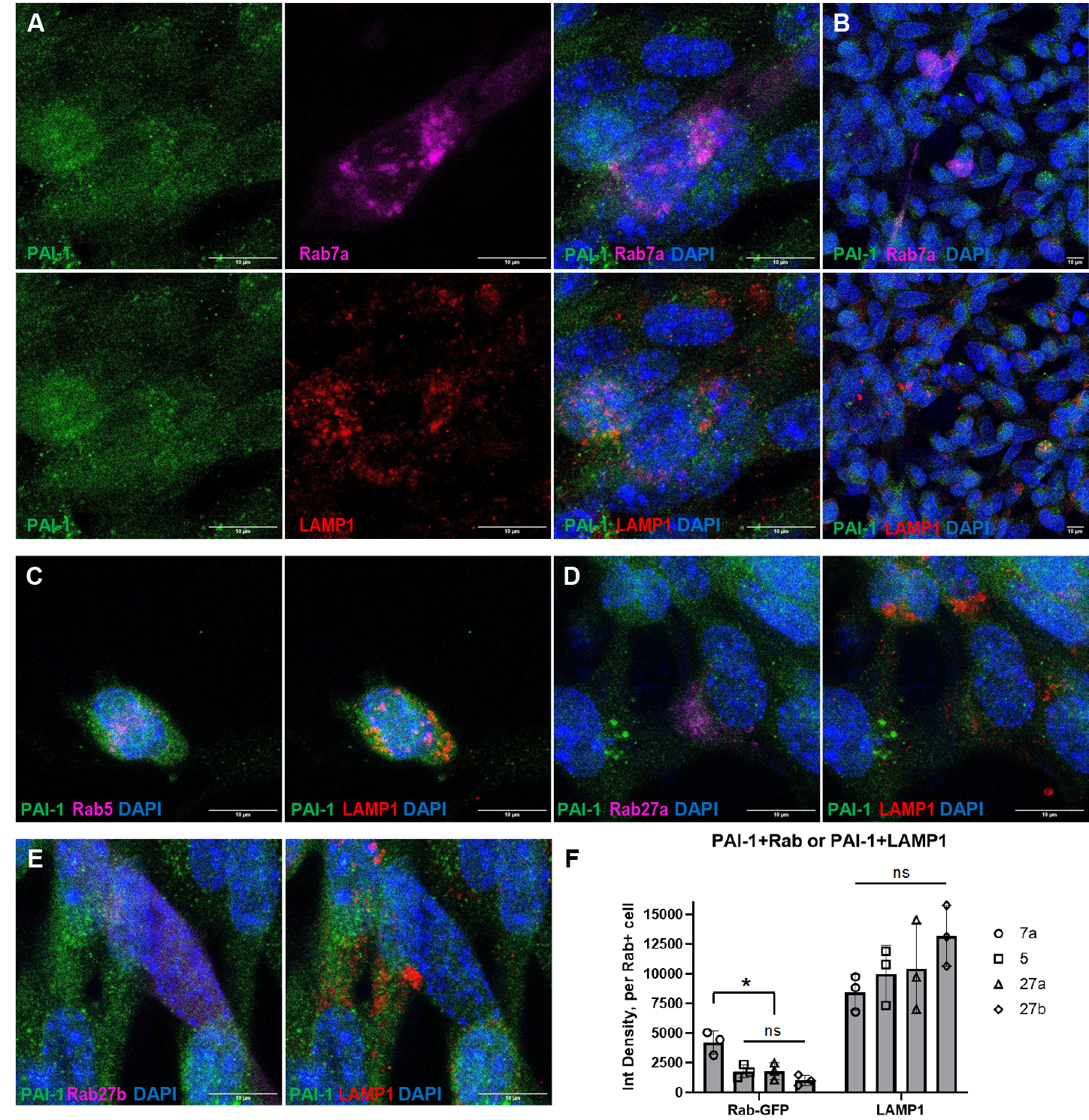

Supplement: Supplementary file 6 — Supplementary Material 6 [file 13046_2025_3473_MOESM6_ESM.tif]

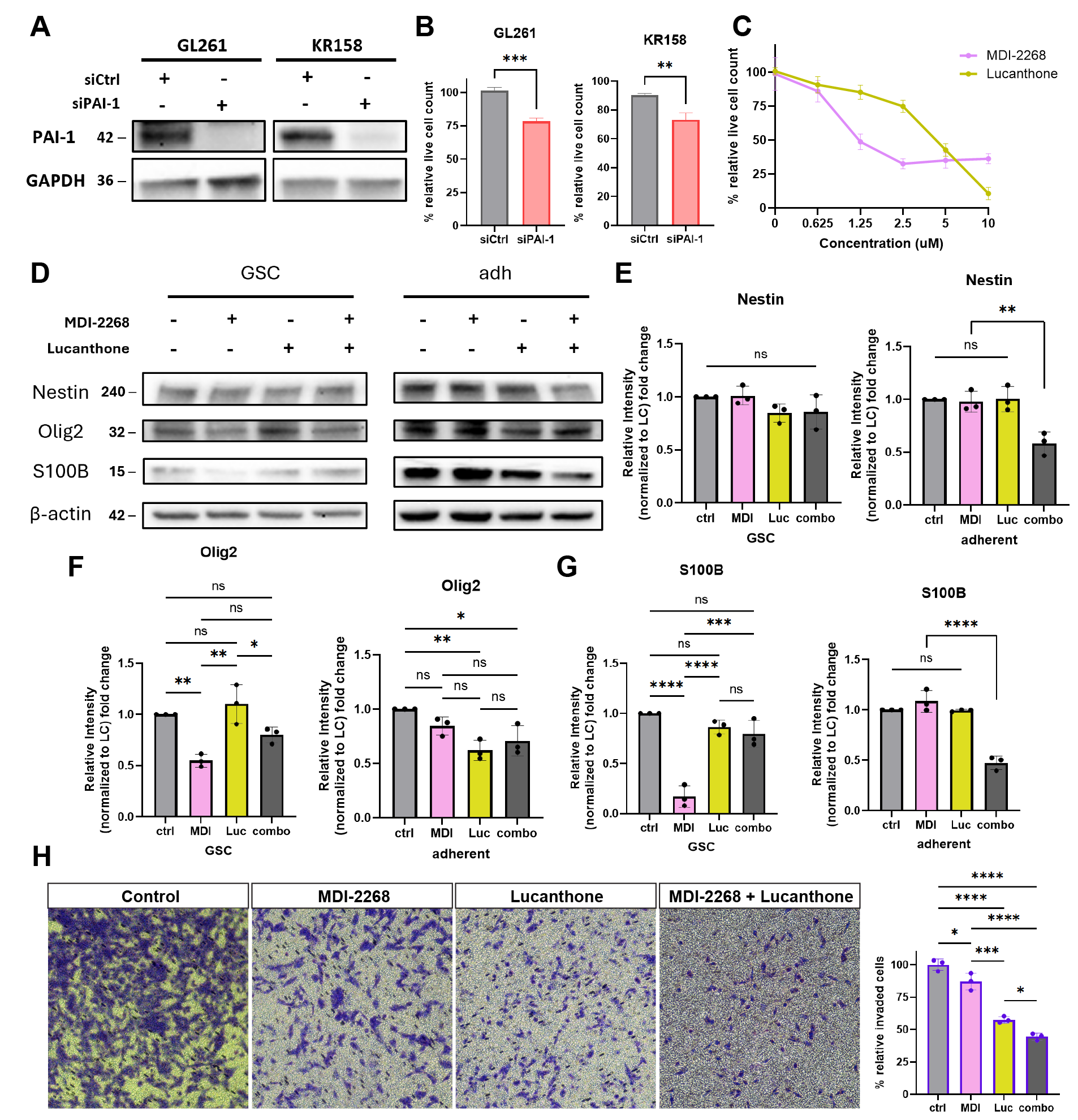

Supplement: Supplementary file 7 — Supplementary Material 7 [file 13046_2025_3473_MOESM7_ESM.tif]

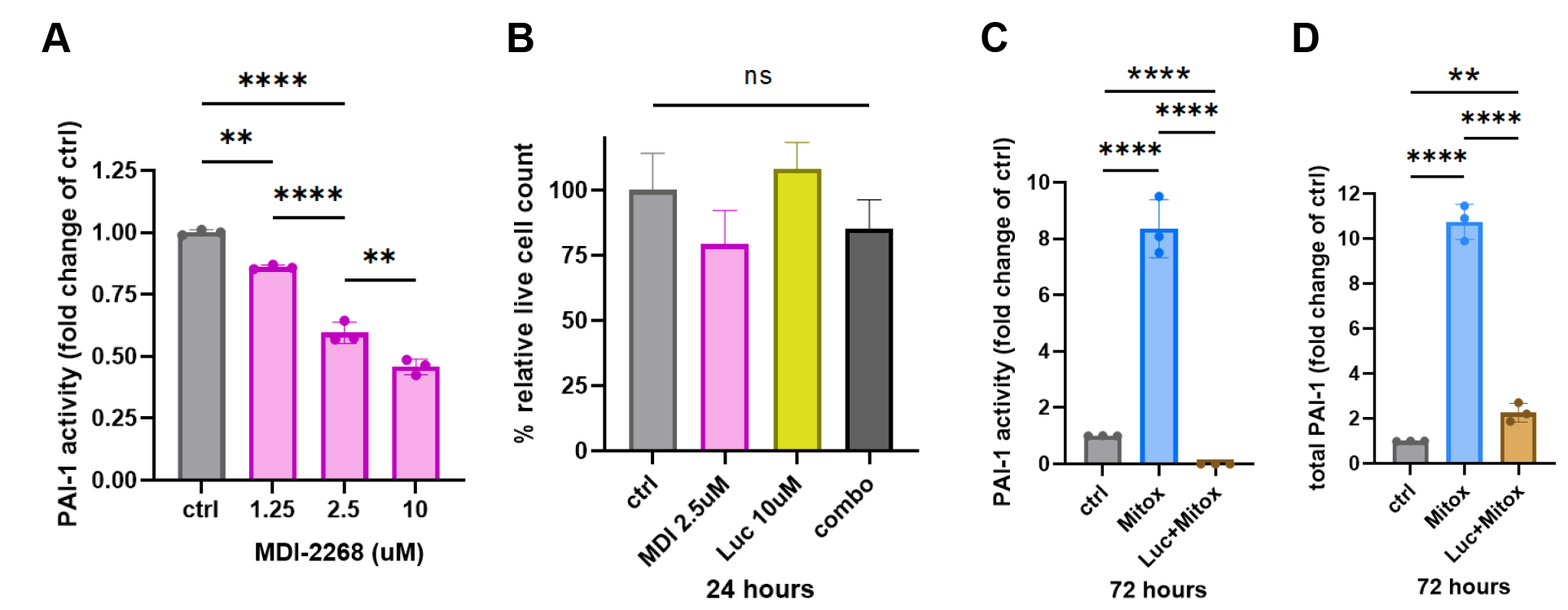

Supplement: Supplementary file 8 — Supplementary Material 8 [file 13046_2025_3473_MOESM8_ESM.tif]

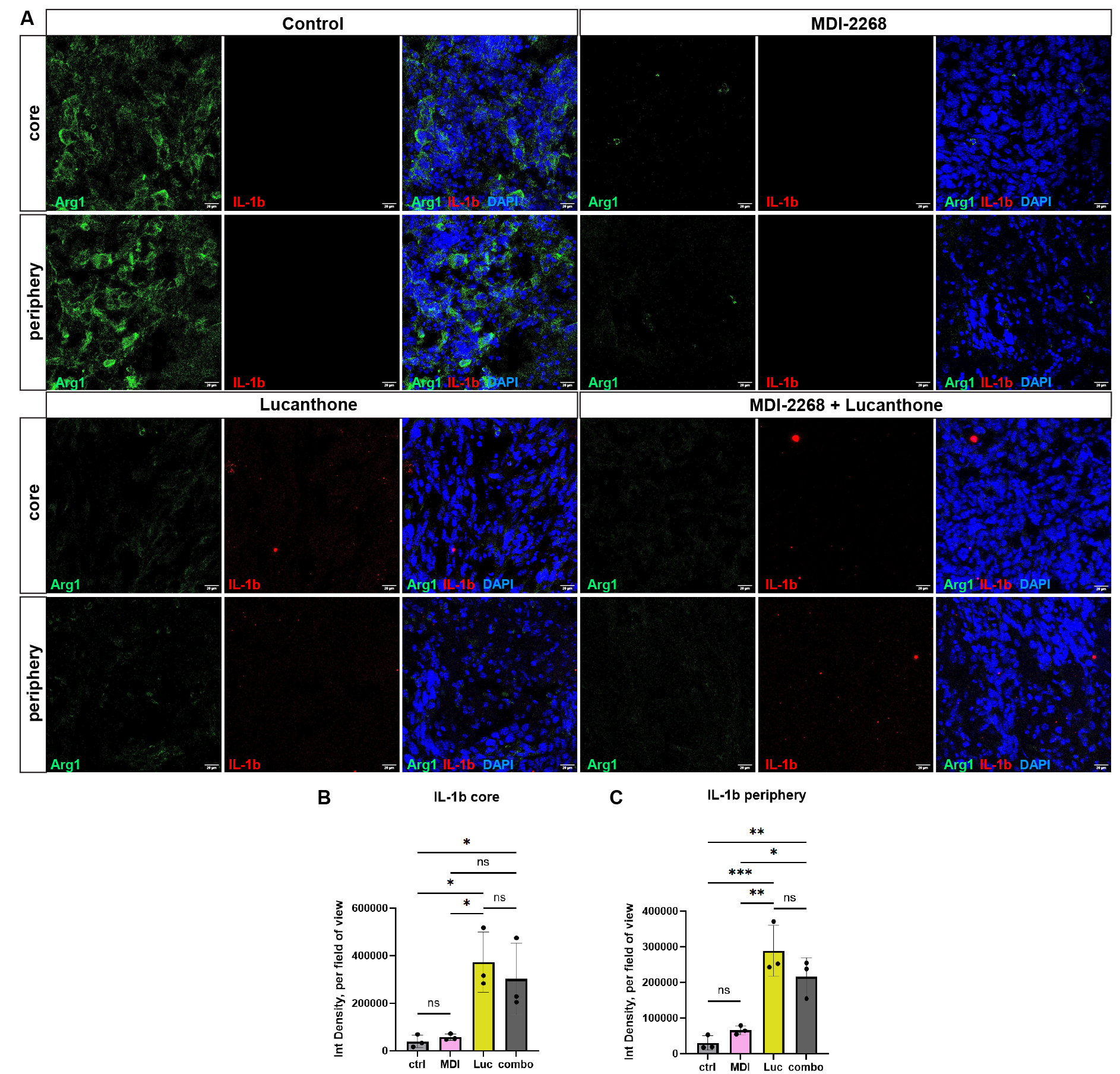

Supplement: Supplementary file 9 — Supplementary Material 9 [file 13046_2025_3473_MOESM9_ESM.tif]

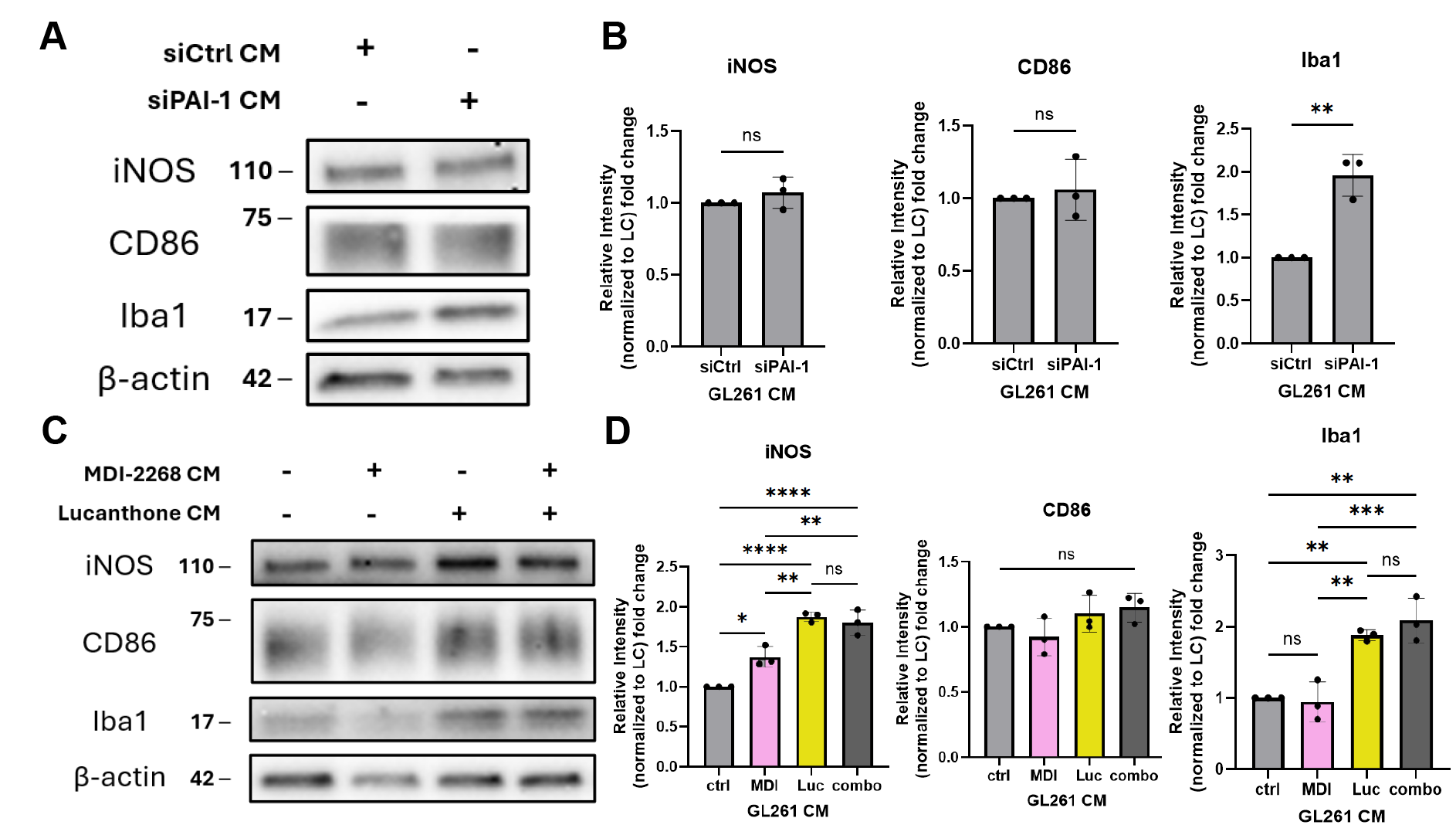

Supplement: Supplementary file 10 — Supplementary Material 10 [file 13046_2025_3473_MOESM10_ESM.tif]
